# Identification of novel miRNAs from drought tolerant rice variety Nagina 22

**DOI:** 10.1038/srep30786

**Published:** 2016-08-08

**Authors:** Roseeta Devi Mutum, Santosh Kumar, Sonia Balyan, Shivani Kansal, Saloni Mathur, Saurabh Raghuvanshi

**Affiliations:** 1 Department of Plant Molecular Biology, University of Delhi South Campus, Benito Juarez Road, New Delhi – 110021, India; 2 National Institute of Plant Genome Research, Aruna Asaf Ali Marg, New Delhi – 110067, India

## Abstract

MicroRNAs regulate a spectrum of developmental and biochemical processes in plants and animals. Thus, knowledge of the entire miRNome is essential to understand the complete regulatory schema of any organism. The current study attempts to unravel yet undiscovered miRNA genes in rice. Analysis of small RNA libraries from various tissues of drought-tolerant *‘aus’* rice variety Nagina 22 (N22) identified 71 novel miRNAs. These were validated based on precursor hairpin structure, small RNA mapping pattern, ‘star’ sequence, conservation and identification of targets based on degradome data. While some novel miRNAs were conserved in other monocots and dicots, most appear to be lineage-specific. They were segregated into two different classes based on the closeness to the classical miRNA definition. Interestingly, evidence of a miRNA-like cleavage was found even for miRNAs that lie beyond the classical definition. Several novel miRNAs displayed tissue-enriched and/or drought responsive expression. Generation and analysis of the degradome data from N22 along with publicly available degradome identified several high confidence targets implicated in regulation of fundamental processes such as flowering and stress response. Thus, discovery of these novel miRNAs considerably expands the dimension of the miRNA-mediated regulation in rice.

MicroRNAs are one of the major components of molecular networks that regulate several plant developmental processes such as leaf development, flowering time, organ polarity^1^ as well as biotic and abiotic stress responses^2^,^3^. Thus, discovery of novel miRNA genes that have not been reported in any organism would have a significant impact on our understanding of the complex molecular regulatory networks. Since discovery of miRNA genes is primarily based on detection of their expression in the small RNA (sRNA) NGS libraries, no single data set is sufficient to identify miRNA genes. Consequently, diverse tissues, growth conditions and varieties need to be explored in order to identify and characterize miRNA genes. Response to abiotic stress is perhaps one of the most widely researched topics due to its impact on the productivity of major agro-economically important crops such as rice, a model monocotyledon. Drought imposes a major threat to its productivity. Water stress during reproductive stage severely affects panicle exsertion and anther dehiscence leading to crop failure^4^. India has a rich collection of rice germplasm wherein many cultivars/varieties have a natural tolerance to various stress conditions. In general, upland rice cultivars, like Nagina 22 (N22), have a better tolerance to drought as compared to rainfed lowland cultivars like Pusa Basmati 1 (PB1). N22 is a deep-rooted, drought- and heat-tolerant^5^,^6^,^7^ rice cultivar. Detailed molecular characterization of such drought-tolerant cultivars would provide valuable information about the nature of adaptive diversity evolved in these cultivars^8^,^9^. N22 offers unique opportunity as a model for studying stress adaptive diversity as it harbors traits, at both physiological^6^ and molecular level^5^,^10^, to combat drought and heat stress. Nevertheless, only a couple of studies have characterized and explored the dynamism of miRNA population in N22^8^,^9^.

Currently, 592 rice miRNA precursor entries are available in the miRBase database release 21. Study with sRNA deep sequenced data in N22 rice further identified a few novel miRNAs^9^, indicating that several miRNA genes remain undiscovered. In general, deep sequenced sRNA populations have been widely used for identification of novel miRNAs in different plant species^11^,^12^,^13^,^14^,^15^,^16^. A major challenge in miRNA gene mining is the presence of small interfering RNAs (siRNAs), which are similar to miRNAs and can only be differentiated by their biogenesis^17^. siRNAs originate from double stranded (ds) precursors formed by intermolecular hybridization of two complementary RNA strands while miRNAs are derived from RNA Pol II transcribed single stranded (ss) precursors possessing a hairpin structure due to intramolecular self-complementarity^17^. The hairpin precursors are processed by Dicer-Like 1 or 4 (DCL1 or DCL4)^18^,^19^ to produce a dsRNA duplex of ~21–24 bp. HASTY transports this duplex out of the nucleus^20^, which is then methylated by HUA enhancer (HEN1) at 3′ end^21^,^22^. The guide or mature strand is incorporated into the RNA induced silencing complex (RISC) whereby the effector protein Argonaute (AGO)^23^ mediates target mRNA identification and cleavage or translational arrest. The other strand of the duplex called the miRNA* (star) is usually degraded. However, there are reports on the functionality of miRNA* as well, under certain conditions^24^,^25^. In order to classify sRNA as a bonafide miRNA, certain criteria based on its secondary structure, expression, biogenesis and conservation need to be considered^26^. Along with the primary criteria of secondary structure, the mapping pattern of the sRNA tags on the genome helps in the identification of the probable miRNA gene locus. A tightly stacked mapping of sRNA tags at the locus is indicative of miRNA processing whereas a dispersed mapping all over the locus is more suggestive of a siRNA-like miRNA^27^.

The main objective of the current study was to identify miRNA genes that have not been reported so far in rice or any other organism. Deep sequencing of the sRNA populations from different tissues such as flag leaf, spikelets ‘heading stage’ and spikelets ‘anthesis stage’ and mature root of N22 plants grown under control and drought conditions was done. Subsequent rigorous analysis, based on well-defined criterion^26^,^28^, identified several putative novel miRNA genes loci. To the best of our knowledge, except an isolated study^9^, sRNA population from these tissues has not been utilized for identification of novel miRNA genes. qRT-PCR analysis was done to validate their expression wherein several of these novel miRNAs were found to have drought responsive as well as tissue preferential expression patterns. While some novel miRNAs were found to be conserved in other plants, most were lineage-specific as they were primarily detected in multiple rice sRNA libraries. The biological activity of these novel miRNAs was confirmed by identifying their target genes and the cut-site with the help of degradome data. Thus, multiple lines of evidences were gathered to support the existence and activity of these novel miRNA genes.

## Results

### Identification of novel miRNAs in N22

Small RNA data is a major resource for identification of novel miRNA genes. Thus, in order to uncover yet unidentified novel MIR gene loci in rice, five sRNA libraries from adult tissues (flag leaf control & stress, heading spikelet control & stress and anthesis spikelet control) of the rice cultivar, N22 were deep sequenced to generate >19 million sRNA tags. This data was used for identification of any unreported miRNA genes as well as to study modulation of miRNome (already known miRNAs) under environmental and temporal cues. While the data on miRNome modulation is presented elsewhere (Balyan S. *et al.*, under-review), the current study summarizes the discovery of novel miRNA genes from the sRNA data. The detailed analysis pipeline has been described in the ‘materials and methods’ section. In summary, all the sRNA tags were mapped to the N22 genome and genomic loci having a non-phasic mapping of sRNA tags were identified. Subsequently, genomic sequences (~200 bp) adjoining these sites were extracted and considered as the putative novel pre-miRNA sequence. These putative novel pre-miRNA sequences were then analyzed for their ability to form a miRNA-like hairpin structure. A total of 2,010 intergenic and 542 intronic putative miRNA loci were initially selected based on hairpin structures. A second round of screening was done to ensure that the sRNA tags within the putative miRNA precursor region clustered at a particular location i.e. mature sequence region^27^. Ultimately, we could identify 71 putative novel miRNA gene loci in N22, details of which are summarized in Supplementary Table S1. Further analysis of these putative novel mature miRNA sequences identified 18 novel miRNAs that had at least 17 bp sequence similarity to miRNAs reported in published literature (Supplementary Table S2) on rice but not available at miRBase database. Thirteen of them (n-032, n-042, n-054, n-064, n-069, n-098, n-102, n-107, n-120, n-128, n-131, n-149 and n-184) did not have significant similarity at the precursor sequence level, while 5 of the putative novel miRNAs (n-020, n-055, n-169, n-050 and n-213) shared appreciable similarity at the precursor level. Only one putative novel miRNA i.e., n-020 was found to be identical with miR2175–0.2^29^ or miR3018^30^, which had been reported separately. In summary, 17 of the putative novel molecules identified in this study appear to be family members of miRNAs reported in the literature while one was found to be the same molecule.

### Characterization and classification of novel miRNAs

The putative novel miRNAs were further analyzed from several aspects in order to validate and characterize them. One of the foremost criteria for defining a miRNA gene is that the precursor transcript should be able to fold back into a typical hairpin structure that has ≤4 symmetrical mismatches within the duplex and/or only 1–2 asymmetric bulge(s) of up to 2 bases^26^. Close scrutiny of the secondary structures indicated that hairpin structures of all the 71 putative novel miRNA candidates conform to the established norms. The secondary structures of the 71 candidates are provided in Supplementary Fig. S1 while their precursor sequences are given in Supplementary Table S1. Further, presence of ‘star’ sequence is another important criteria for identification of miRNA genes. However, due to unstable nature of the sequence it is difficult to detect the ‘star’ sequence for all the miRNA genes. We were able to detect the ‘star’ sequence for 36 novel miRNA genes (Supplementary Table S1).

**Figure 1:**
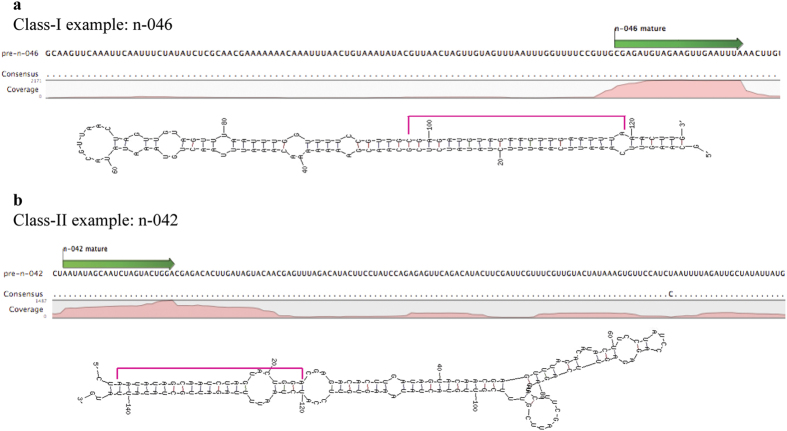
Classification of novel miRNA candidates. Examples of 2 different classes of novel miRNA molecules. Mapping patterns (‘pink’ region) of sRNA tags on the precursor sequences of novel miRNAs and respective secondary hairpin foldbacks generated using UNAFold software for (**a**) n-046 (class-I) and (**b**) n-042 (class-II).

Another important characteristic feature of a miRNA gene locus is the manner in which sRNA tags map on the precursor sequence. Since the generation of the mature miRNA population from the precursor sequence is through a near precise cut made by DCL1 and associated proteins, majority of the mature miRNA tags share almost a common 5′ end (with ±3 bases variation)^31^. The 3′ end, however is quite variable. Thus, when sRNA tags are mapped on the precursor sequences, most of them should map as a tight cluster in the region that is proposed to give rise to the mature miRNA sequence. Scrutiny of the mapping pattern of sRNA reads in the entire precursor sequence indicated that majority of tags mapped in the proposed mature miRNA region. However, in some cases the mapping of tags was slightly dispersive in nature. Based on the mapping pattern, the putative novel miRNAs were divided into two classes. While both classes had a good hairpin secondary structure, class-I loci had a consistent mapping pattern (≥40% of total reads mapped to the mature sequence region) while class-II loci had slightly dispersive mapping pattern (<40% of total reads mapped to the mature sequence region). The percentage of sRNA reads abundance at mature position as compared to the total reads mapped to the precursor is given in Supplementary Table S1. Examples of each class based on mapping pattern and secondary structure is given in Fig. 1a,b. In summary, there are 40 class-I and 31 class-II novel miRNA candidates.

**Figure 2:**
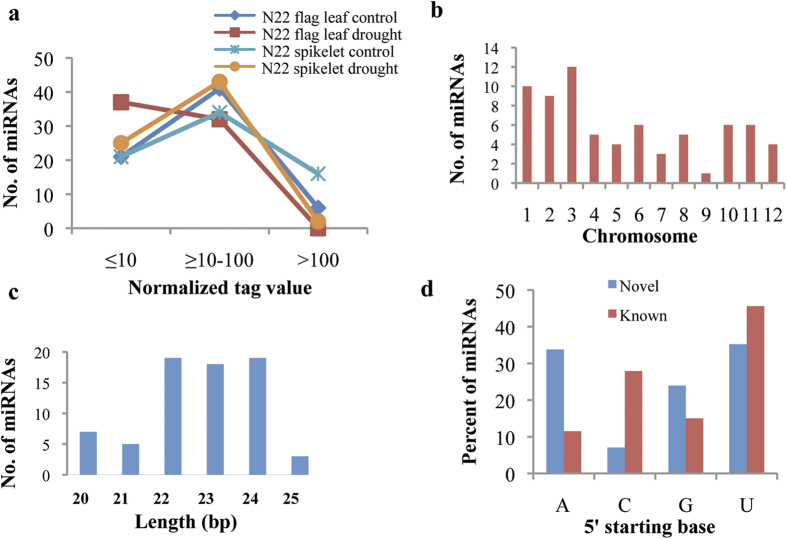
Characterization of novel miRNAs in N22. (**a**) Comparison of normalized tag abundance of novel miRNAs in different small RNA libraries of N22. (**b**) Chromosomal distribution and (**c**) Size distribution of 71 novel miRNAs. (**d**) Comparison of 5′ nucleotide of novel and known miRNAs.

The putative novel miRNAs were also characterized by assessing their abundance in multiple rice sRNA libraries as well as by detecting the mature miRNA sequence with the help of qRT-PCR. As expected, all the novel miRNAs were detected in multiple sRNA libraries of N22 and comparison of their normalized tag abundance in these libraries indicated that most of them express at a moderate level (Fig. 2a). The qRT-PCR based gel profiles of the amplified mature novel miRNA sequences were also generated in order to detect their expression as well as to confirm their size (Supplementary Fig. S2). The amplified product includes the adapter (~100 nt) as well as the mature miRNA (~20 nt) thereby giving the size of the amplified product in the range of ~120 bp. Based on mature sequence length, 49 out of 71 are of canonical length (20–23 bp) while 22 are long molecules (24–25 bp). They are further sub-classified as either intergenic (39) or intronic (32) based on their genomic location. Chromosomal distribution of the 71 novel miRNAs indicates highest abundance at chromosome (chr) 3 followed by chr 1 (Fig. 2b), while size distribution indicates that 22–24 bp novel miRNAs were of highest abundance (Fig. 2c).

Further, association of the miRNAs with the RISC complex is an indication of their functionality, since only functional miRNAs tend to get loaded onto the RISC complex. Thus, publicly available AGO pulldown sRNA libraries published earlier^32^,^33^ were analyzed wherein a significant number (67) of novel miRNAs were found to be associated with the RISC complex (Supplementary Table S3). Tags of 36 out of 67 were found in various AGO1 pulldown libraries while tags of all the 67 novel miRNAs were present in various AGO4 pulldown libraries (Supplementary Table S3). Moreover, sRNAs with different 5′ starting base of the mature sequence have differential AGO loading^32^,^33^,^34^,^35^. The distribution of 5′ starting base in novel and known miRNAs was quite comparative wherein novel miRNAs and known miRNAs (miRBase) had 35.21% and 45.58% with 5′U, 23.94% and 15.01% with 5′G, 33.80% and 11.50% with 5′A and 7.04% and 27.91% with 5′C, respectively (Fig. 2d).

**Figure 3:**
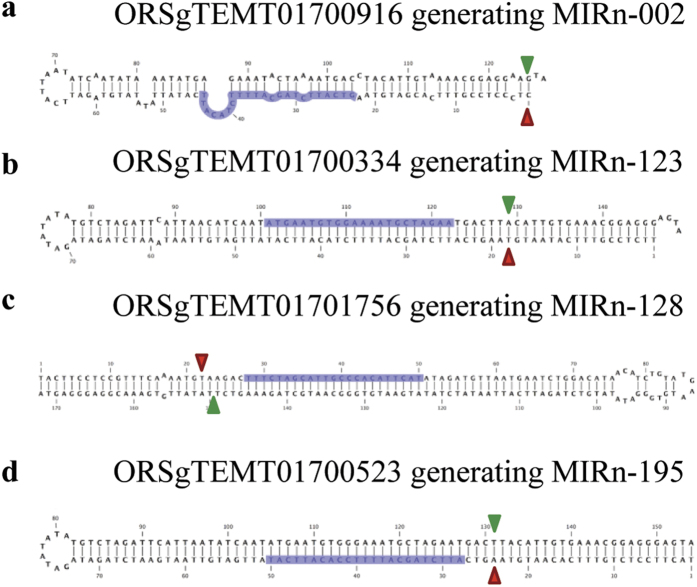
Secondary hairpin structures generated from rice repeat sequences. Secondary hairpin foldbacks of repeats (**a**) ORSgTEMT01700916, (**b**) ORSgTEMT01700334, (**c**) ORSgTEMT01701756 and (**d**) ORSgTEMT01700523. The highlighted portions indicate the mature sequences of the corresponding miRNAs. The start and end of MIR precursor sequences are demarcated by green and red arrowheads, respectively.

Finally, the copy number of all the 71 novel MIR genes was also checked to ascertain if they originated from multiple loci or discrete genomic locus. The study was done by analyzing the occurrence of precursor as well as the mature novel miRNA sequences on the rice genome sequence. Based on the analysis of the precursor sequences, almost all the novel MIRs was found to originate from a single genomic locus except n-170, which was present in 2 copies on the genome. On the other hand, analysis with the mature sequence indicated that, most of the novel miRNAs had <20 genome hits, while 20 of them showed multiple hits (>20) to the genome (Supplementary Table S4). Similar analysis with known rice miRNAs from published literature revealed that many of them had >20 genome hits as well (Supplementary Table S4). Further, since recent studies have also indicated that miRNAs may also originate from within transposable elements (TE)^36^, we explored the rice repeat sequences (RGAP database; www.rice.plantbiology.msu.edu) for the presence of any novel miRNA precursors. Consequently, four novel miRNA candidates, all belonging to class-II i.e., n-002, n-123, n-128 and n-195 were found to have 100% sequence similarity with MITE-adh, type D-like repeats ORSgTEMT01700916, ORSgTEMT01700334, ORSgTEMT01701756 and ORSgTEMT01700523, respectively. The secondary structures of these repeat sequences are shown in Fig. 3a–d.

### Novel miRNA family members

The mature sequences of the novel miRNAs were further analyzed to ascertain if they can be clubbed as miRNA family members. Based on sequence similarity, 10 novel MIR families containing 27 novel miRNAs were identified. In general, family members share mature sequence similarity and often target related genes. The 10 novel miRNA families thus identified are MIRn-130, MIRn-050, MIRn-059, MIRn-098, MIRn-099, MIRn-102, MIRn-129, MIRn-134, MIRn-160 and MIRn-195. The multiple alignments of their mature sequences are shown in Supplementary Fig. S3a–j. Family of MIRn-160 had the highest number of miRNA members namely, n-160, n-100, n-225, n-169 and n-213.

Additionally, analysis on genomic arrangement of novel miRNAs *vis-à-vis* known miRNAs in N22 genome revealed an interesting fact about a novel miRNA locus. Although present on different chromosomal loci, the precursor of n-026 (chr 5) is very similar (>80%) to that of osa-miR5788 (chr 3). However, their mature sequences have only 5 bp overlap and thus they cannot be classified as family members. Nevertheless, there appears to be some kind of evolutionary relationship between the two miRNAs since their precursor sequences share significant similarity.

### Identification of unreported putative miRNA orthologs in N22

The phylogenetic conservation of miRNA sequences (mature and precursor) as well as the secondary structure facilitates identification of putative orthologous miRNAs^26^. Thus, in addition to the aforementioned novel miRNA genes which were not reported in rice or any other organism, analysis was also done to identify rice orthologs of miRNA genes known in other plant species. Accordingly, as depicted in the analysis pipeline (Supplementary Fig. S4), 22 non-rice known MIR precursors could be aligned with ≥50% sequence similarity to the N22 genome. Of these, 16 had ≤3 mismatches in the mature region. The pairwise alignments of the parent MIR precursors (miRBase) to that of identified rice putative ortholog precursors are shown in Supplementary Fig. S5. Subsequently, secondary structure analysis indicated that rice ortholog of 6 i.e. MIR5056, MIR5368, MIR6187, MIR6196, MIR6221 and MIR9774 had a good hairpin structure while others (MIR6199, MIR5141, MIR9780, MIR6485, MIR6207, MIR6206, MIR6183, MIR2916, MIR7767 and MIR6194) had inconsistent structures, despite a significant conservation at the precursor sequence level. The details of 16 putative orthologs and their secondary structures are summarized in Table 1 and Supplementary Fig. S6a–p, respectively.

### Novel miRNAs are lineage-specific

**Figure 4:**
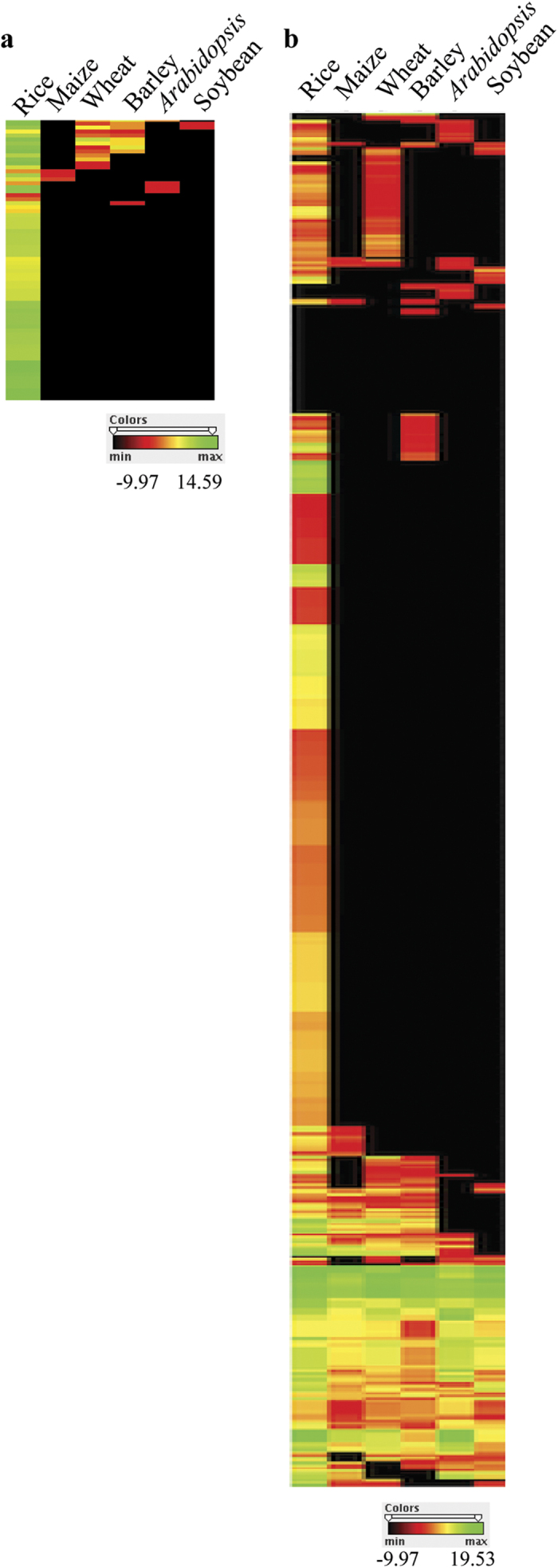
Lineage-specific nature of novel miRNAs. Heat maps showing detection of (**a**) 71 novel and (**b**) known rice miRNAs (miRBase) in sRNA libraries of monocots (rice, maize, wheat, barley) and dicots (*Arabidopsis* and soybean). Color bar indicates the log_2_ transformed values. (Refer text for library details).

In order to check the extent of conservation/divergence of novel miRNAs, their abundance was analyzed in other publicly available sRNA libraries of rice and other plants. A total of 14 sRNA libraries from rice (3), maize (1), wheat (2), barley (3), *Arabidopsis* (3) and soybean (2) available at NCBI ‘SRA’ database were utilized (Supplementary Table S5). Tag abundance of 71 miRNAs could be detected in all the 3 rice sRNA libraries, indicating the reliability for their existence and expression in other varieties/stage/tissue of rice. Further, sRNA tags corresponding to 14 novel miRNAs were detected in other monocots, which include 13 in wheat, 10 in barley and 3 in maize. Heat map of the 71 novel miRNAs in sRNA libraries of rice, maize, wheat, barley, *Arabidopsis* and soybean is shown in Fig. 4a. Similarly, sRNA tags for 6 novel miRNAs were detected in dicots including 4 in *Arabidopsis* and 2 in soybean. While most of them were detected at low levels, some had >5 tags (Supplementary Table S5). Analysis of the genomic loci in the respective plants revealed that the precursors of 3 novel miRNAs in barley (n-006, n-107 and n-118) and 3 in wheat (n-024, n-032 and n-107) had acceptable miRNA-like hairpin structures (Supplementary Fig. S7a–f). Similar analysis for known miRNAs to compare the extent of conservation/divergence indicated that 23.7%, 31.6%, 27.8%, 21.7% and 19.9% of known miRNAs were detected in the sRNA libraries of maize, wheat, barley, *Arabidopsis* and soybean, respectively (Fig. 4b, Supplementary Table S5). In summary, most of these novel miRNAs are considerably conserved in rice whereas only few could be detected in other plants indicating a lineage-specific nature^17^.

### Identification of novel miRNA targets

To ascertain the functionality of these novel miRNAs, ‘degradome sequencing’^37^ or ‘PARE’ (Parallel Analysis of RNA Ends)^38^ was performed to identify and validate target mRNA cleavage. Sequencing of three degradome libraries from anthesis spikelets (ASp), heading spikelets (HSp) and flag leaf (FL) of N22 generated more than six million reads from each library (Supplementary Table S6). Subsequent analysis of the degradome data by CleaveLand analysis pipeline^39^ identified 40:105, 20:52 and 30:91 novel miRNA:target pairs in the PARE libraries from ASp, HSp and FL, respectively. CleaveLand analysis was further extended by including five more publicly available rice PARE libraries (SRR032098, SRR039716-039720, SRR521269, SRR032097 and SRR034102) (Supplementary Table S7). In summary, analyses of data from 8 PARE libraries indicated putative multiple targets of the novel miRNAs (including low confidence targets). Distribution of miRNA:target pairs based on different confidence parameters (degradome read number, category and P-value) of CleaveLand pipeline is shown in Supplementary Table S8. Analysis of the top ten targets for each novel miRNAs reveals diverse targets including transcription factors, metabolic enzymes, signaling components (kinases and phosphatases) that have been implicated in important cellular processes such as plant development and stress (biotic and abiotic) response (Supplementary Table S9).

**Figure 5:**
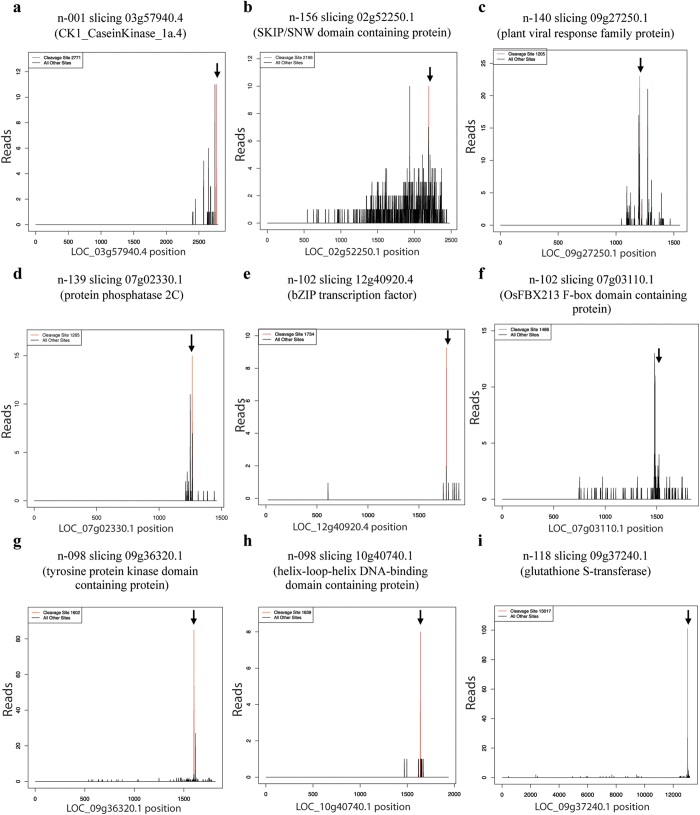
T-plots of novel miRNA targets. T-plots of high confidence targets of selected novel miRNAs. The downward arrow indicates the cleavage site. (**a**) n-001 slicing 03g57940.4 (CK1_CaseinKinase_1a.4) at 2771. (**b**) n-156 slicing 02g52250.1 (SKIP/SNW domain containing protein) at 2196. (**c**) n-140 slicing 09g27250.1 (plant viral response family protein) at 1205. (**d**) n-139 slicing 07g02330.1 (protein phosphatase 2C) at 1267. (**e**) n-102 slicing 12g40920.4 (bZIP transcription factor) at 1734. (**f**) n-102 slicing 07g03110.1 (OsFBX213-F box domain containing protein) at 1486. (**g**) n-098 slicing 09g36320.1 (tyrosine protein kinase domain containing protein) at 1602. (**h**) n-098 slicing 10g40740.1 (helix-loop-helix DNA-binding domain containing protein) at 1639. (**i**) n-118 slicing 09g37240.1 (glutathione S-transferase) at 13017.

Of all the putative targets, essentially 33 miRNA:target pairs (25 genes targeted by 19 novel miRNAs) are of high confidence (degradome reads ≥10, category ≤2 and P-value ≤0.05) (Table 2). A cut-off based on simply the alignment score was not considered as it has been reported that miRNAs could cleave targets even with higher mismatches^40^. T-plots (target plots) for selected high confidence and important targets of novel miRNAs are shown in Fig. 5a–i and discussed in subsequent text. Of these 19 novel miRNAs having high confidence target genes, 11 were of class-I while 8 of class-II. Further, novel miRNAs with multiple loci also had high confidence targets based on the degradome data indicating their biological activity. For example, novel miRNA n-121 with 397 genomic hits (at mature level) had a target identified with 36 degradome reads (category ‘0’). Similarly, n-118 having 177 genome hits had a target supported by 101 degradome reads (category ‘0’).

Subsequently, targets of orthologous miRNAs were also identified from the 8 PARE libraries (Supplementary Table S10). Targeting of 38 genes by 10 orthologous miRNAs are supported by ≥10 degradome reads (Supplementary Fig. S8a) while 11 pairs (4 miRNA:11 target genes) are of ≥10 degradome reads and category ‘0’. Interestingly, two (osa-miR6485 and osa-miR6207) of the miRNA:target pairs with ‘0’ category do not have a consistent hairpin structures. The targeting of LOC_Os11g15240 (retrotransposon) by osa-miR9774 could be supported with highest confidence. Representative ‘t-plots’ for some of the orthologous miRNA targets are shown in Supplementary Fig. S8b–m.

The degradome libraries were also used to identify targets of known miRNAs. Consequently, 266:895, 266:925 and 350:3054 miRNA:target pairs were obtained from N22 ASp, HSp and FL, degradome libraries, respectively (Supplementary Table S6). On further inclusion of 5 more PARE libraries, 551:16,381 miRNA:target pairs were identified (no filters applied on degradome criteria). Out of this, 102:126 miRNA:target pairs are of high confidence (≥10, category ≤2 and P-value ≤0.05) (Supplementary Table S7). The ratio of high confidence targets among all the targets is quite comparable for both novel and known miRNAs (Supplementary Tables S9 and S10).

### Expressions of novel miRNAs in response to developmental and environmental cues

Expressions of miRNAs are guided by environmental or developmental cues. Thus, sRNA deep sequenced data from various tissues of N22 (FL, HSp, ASp and MR, i.e. mature root) grown under control and drought conditions was analyzed to assess the tissue-biased and drought responsive nature of the novel miRNAs. Analysis of the sRNA tags from different tissues of plants grown under control conditions identified tissue preferential expression of several novel miRNA genes. In summary, 47, 51, 46 and 49 novel miRNAs were found to have ≥10 normalized tag density (RPM) in FL, HSp, ASp and MR of N22. Further, 5 novel miRNA (n-024, n-039, n-063, n-148 and n-153) were significantly up-regulated (fold change ≥4, P-value ≤0.05) in the FL as compared to both HSp and MR whereas 8 novel miRNAs (n-001, n-019, n-028, n-046, n-050, n-130, n-140 and n-173) were up-regulated in HSp as compared to both FL and MR (Supplementary Table S11). Incidentally, we did not find any novel miRNA that was significantly up-regulated in MR as compared to both FL and HSp. Comparison of the spikelet from the two stages of inflorescence development i.e. heading and anthesis identified 28 novel miRNAs that were differentially regulated even in these closely spaced developmental stages (Supplementary Table 11). While 19 novel miRNAs were up-regulated, 9 were down-regulated in the spikelets during transition from heading to anthesis. An interesting member of this group is n-001 which is significantly down-regulated (42 folds) in ASp and targets a Casein Kinase-1 protein, “Hd16” (*HEADING DATE 16*) or “El1” (Early flowering 1)^41^,^42^.

**Figure 6:**
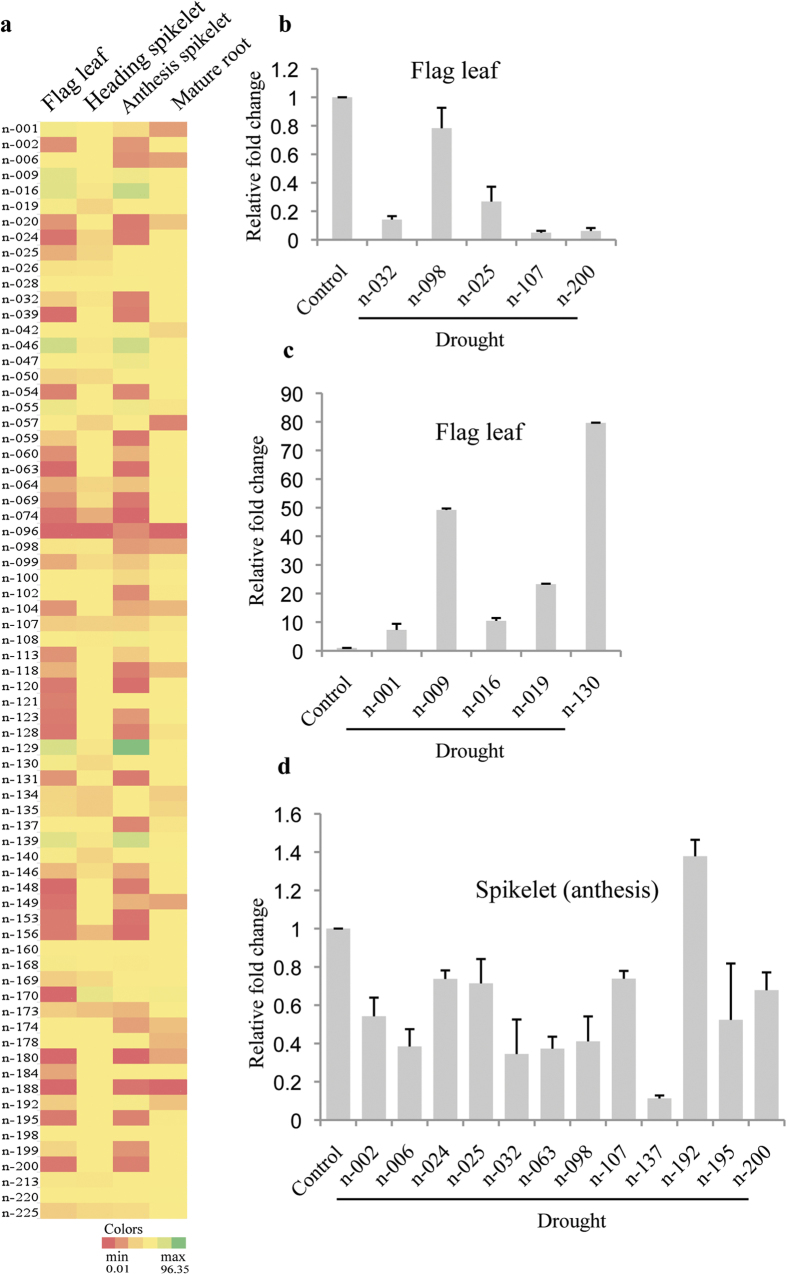
Drought responsive expression analysis of novel miRNAs in N22. (**a**) Heat map of 71 novel miRNAs based on fold change values in FL, HSp, ASp and MR upon drought stress. qRT-PCR expression profiling for novel miRNAs (**b**) down-regulated in flag leaf and (**c**) up-regulated in flag leaf during drought. (**d**) Novel miRNAs expression in anthesis spikelet during drought.

Further, comparison of the sRNA libraries from control and drought treated N22 plants revealed several drought responsive novel miRNAs. In total, 48 novel miRNAs were found to be significantly drought stress responsive in the four different tissues of N22. Heat map showing the fold change expression of 71 novel miRNAs in FL, HSp, ASp and MR in drought stress treated libraries as compared to control libraries are shown in Fig. 6a. Most of the novel miRNAs were down-regulated while only 8, 1, 8 and 1 novel miRNAs were up-regulated in FL, HSp, ASp and MR, respectively (Supplementary Table S12). Interestingly, four novel miRNAs *viz*., n-016 and n-129 in ASp and n-046 and n-170 in FL had ≤1 (normalized) tag under control conditions but ≥30 tags (normalized) during drought.

The drought-regulated expression of several novel miRNAs which target important genes was validated by qRT-PCR (Fig. 6b–d). Novel miRNAs n-025, n-032, n-098, n107 and n-200 were found to be down-regulated (Fig. 6b) whereas n-001, n-009, n-016, n-019 and n-130 were up-regulated in the FL during drought (Fig. 6c). Similarly, except for n-192, miRNAs n-002, n-006, n-024, n-025, n-032, n-063, n-098, n-107, n-137, n-195 and n-200 were down-regulated in ASp during drought (Fig. 6c). Novel miRNAs n-025, n-032, n-098, n-107 and n-200 were commonly down-regulated in both FL and ASp during drought. We further verified drought responsiveness of the target genes of these drought responsive novel miRNAs. Indeed, analysis of the transcriptome data (Indica Rice Database; www.genomeindia.org.in/irdb) revealed that targets of many of the novel miRNAs showed anti-correlation in expression during drought stress (Supplementary Table S13).

The sRNA libraries were also used to assess the expression of the putative orthologous miRNAs. The expression profile of 17 mature putative orthologous miRNAs is depicted by heat map generated from transformed values of sRNA abundance in 8 different N22 sRNA libraries (Supplementary Fig. S8n). Five of them (osa-miR5368, osa-miR6485, osa-miR6196, osa-miR2916 and osa-miR9774) were constitutively expressed in most of the N22 libraries while expression of osa-miR6187, osa-miR6199, osa-miR6206, osa-miR6221-5p, osa-miR6221-3p and osa-miR9780 could not be detected in our libraries.

## Discussion

MicroRNAs regulate various metabolic and developmental processes^43^,^44^ and thus knowledge of the complete repertoire of the miRNA genes is critical to understand the complicated regulatory mechanisms in both plants and animals. Discovery of miRNA genes has been revolutionized by next generation sequencing technologies. In rice, over 700 mature miRNAs (miRBase release 21) have been identified, nevertheless, the list is not saturating. Like any other gene, expression of miRNAs is under tight control of spatial, temporal and environmental cues. Since identification of miRNA is primarily based on the detection of its expression (e.g. RNA seq), low expressing miRNAs or those having restricted expression may be missed if appropriate sRNA libraries are not available. In the current study, we have explored sRNA libraries from flag leaves, spikelets and roots tissues of a drought tolerant *‘aus’* rice variety Nagina 22 and after rigorous multi-tiered analysis were able to identify 71 novel miRNA genes that have not been reported earlier in rice or any other organism. Interestingly, 39 of them were intergenic while 32 were localized within the introns of protein coding genes. Four of the novel miRNAs were found to originate from within the repeat sequences in rice. Earlier studies have also reported miRNAs originating from repetitive regions of genome^36^. MITEs (Miniature Inverted Transposable Elements) loci in *Arabidopsis* and rice have been reported to generate sRNAs^45^. Moreover, some of these novel genes are probably family members of already known miRNA genes in rice while others could be grouped as members of novel miRNA families. Family members represent similar/same mature sequence but with distinct MIR precursor genes^46^. They probably have a common evolutionary origin and often cleave same/related target genes.

In order to confirm the existence of these novel miRNAs, multiple lines of evidences were investigated. These included acceptable hairpin structure^27^,^28^, mapping pattern of sRNA tags on the precursor, qRT-PCR based expression, detection of ‘star’ sequence, conservation in sRNA libraries from rice and other plants as well as identification of target genes based on degradome data. In general, all the novel miRNAs had hairpin structure similar to a typical miRNA gene and could be detected by both sRNA NGS data as well as qRT-PCR analysis. Thus, there is a high level of confidence in these novel miRNA genes. Detection of these novel miRNA genes in the AGO pulldown sRNA libraries further supports their existence and functionality. In-depth characterization of these novel miRNAs indicated that while all had a typical miRNA hairpin structure, the mapping pattern of sRNA tags on some novel miRNA precursors was not very stacked, indicating an imprecise processing. Thus, we segregated the novel miRNAs based on the mapping patterns wherein class-I genes can be referred to as typical miRNA genes whereas the class-II genes are more of a siRNA-like miRNA genes^27^. Several earlier studies have also reported existence of such siRNA-like miRNA loci^27^,^47^. Interestingly, it was possible to identify high confidence targets (with typical miRNA like precise target cleavage) for several class-II novel miRNA genes indicating functionality of the siRNA-like miRNA genes. Thus, even though class-II genes undergo an imprecise processing, they are able to cleave the target in a typical miRNA like manner. Similarly, it was possible to identify high confidence targets even for novel miRNA genes that have multiple genome hits (>20). Incidentally, some known miRNA genes (deposited in the miRBase) are also known to have more than 20 genome hits. For example, miR818 has 472 hits, miR815 has 87 hits in rice genome. Apparently, although the mature sequence may hit the genome multiple times, however, their corresponding precursor sequences may not be able to fold into a typical miRNA like hairpin.

Another important feature of miRNA genes is that most loci are conserved across organisms. However, non-conserved miRNAs that are weakly expressed and have limited phylogenetic conservation are a universal feature of land plants^48^. While, almost all the novel miRNAs could be detected in the publicly available rice (mostly *japonica*) sRNA libraries, only few were conserved in other monocots such as wheat, maize and barley. A couple of novel miRNAs were even conserved in dicots (*Arabidopsis* and soybean) as well. Thus, apparently most of the novel miRNAs were lineage-specific (rice) in nature and may have recently evolved^17^.

An interesting feature of the miRNAs, in general, is the distribution of the first 5′ base of the mature miRNA sequence. In plants, miRNAs and 21 bp siRNAs with 5′ U are primarily directed to AGO1, 24 bp siRNAs with 5′ A onto AGO4, sRNAs with 5′ A on AGO2 and those with 5′ C are primarily loaded onto AGO5^32^. The distribution of the 5′ base of the novel miRNAs identified in the study was quite similar to known miRNAs. Further, number of AGO4 associated novel miRNAs was comparatively higher than AGO1 associated miRNAs. Indeed, many of these miRNAs could be defined as long miRNAs having length ≥24 bp. Studies on these miRNAs would yield interesting results since long miRNAs are known to be involved in regulating DNA methylation^33^.

Besides basic identification and characterization of the novel miRNA genes, the evidence of the cleavage of the target transcript is one of the most convincing indications of a miRNA activity. Thus, analysis of the degraded transcripts is essential in order to validate the existence of the novel miRNA genes as well as to assess the biological impact of the novel miRNA activity. Consequently, analysis of in-house generated degradome libraries as well as several publicly available degradome libraries of rice^32^,^49^,^50^,^51^ revealed appreciable number (≥10) of degradome tags for more than 50% of the novel miRNAs. Many of these targets are of high confidence and thus reinforce the biological functionality of these novel miRNA genes. A global overview of all the targets of these novel miRNAs indicates a wide range of biological activities ranging from gene regulation to metabolic pathways. Many of these targets are components of biochemical pathways (RiceCyc, http://pathway.gramene.org/gramene/ricecyc.shtml). For example, *GLUTATHIONE S-TRANSFERASE* targeted by n-118 is involved in ROS metabolism whereas *ARGINYL-TRNA SYNTHETASE* targeted by n-042 (class-II) is involved in ‘tRNA charging’ (Metabolic cluster pathway) wherein it catalyses the formation of L-arginyl tRNA from L-arginine with the use of an ATP molecule. Similarly, *POLYGALACTURONASE* targeted by n-173 (class-I) is involved in ‘homogalacturonan degradation pathway’*. CYCLOPROPANE-FATTY-ACYL-PHOSPHOLIPID SYNTHASE* targeted by n-108 (class-II) is involved in ‘Fatty acids and lipids biosynthesis pathway,’ specifically in cyclopropane and cyclopropene fatty acid biosynthesis. Other important targets include *PROTEIN PHOSPHATASE 2C*, *SKIP/SNW* protein, plant viral response protein, *F-BOX* domain containing protein, *TYROSINE PROTEIN KINASE* targeted by n-139, n-156, n-140, n-102, and n-098, respectively.

The study further explores the expression dynamic of these miRNAs during the reproductive phase (‘heading’ and ‘anthesis’) of the rice plant development and includes tissue such as flag leaf, spikelets and roots, which have not been explored extensively in terms of miRNA expression dynamics. Flag leaf, which bears the panicle, is the major source of photosynthates to the developing panicle and thus a very important tissue^52^,^53^. Novel miRNA n-024 and n-063, which putatively target a ‘*GLUTATHIONE S-TRANSFERASE’* and an *‘EARLY FLOWERING PROTEIN’*, were found to be significantly enriched in the flag leaf as compared to other tissues. Similarly, roots are important as they are the first to experience change in the soil moisture content. Studies on comparative proteome studies in the roots of wild type and *DREB1A* over-expression lines under drought stress conditions indicated that many stress and defense related proteins were up-regulated in both plants but a novel protein R40C1 was up-regulated only in transgenic roots^54^. Root-specific over-expression of *OsNAC10* increases grain yield significantly under field drought conditions by enlarging roots and enhancing drought tolerance^55^. More than 70% of all the novel miRNA genes expressed appreciably in the mature roots. Under drought conditions almost all novel miRNAs were found to be down-regulated in the mature roots, except n-170 that gets up-regulated and putatively targets an ‘*AUXIN RESPONSE FACTOR (ARFs)’.* Other known miRNAs such as miR160 and miR167, which also target ARFs are known to be involved in the regulation of root development^56^,^57^.

Another interesting developmentally regulated novel miRNA is n-001 that targets “Hd16”. Hd16 is known to phosphorylate rice DELLA protein SLR1 and Ghd7, a CO-like protein which suppresses flowering by inhibiting the expression of Ehd1 (a floral activator)^41^,^42^. Hd16/EL1 is a flowering repressor identified from a cross between Nipponbare and Koshihikari. It is postulated that Hd16 might act as a mediator between floral transition and other developmental processes such as gibberellin signaling and tillering. Similarly, the drought responsive novel miRNAs target several important genes such as *GLUTATHIONE S-TRANSFERASE* (n-024), *GLUTAREDOXIN 2* (n-137), *EARLY FLOWERING PROTEIN* (n-063)*, SKP1-LIKE PROTEIN 1B* (n-192), *GPI-ANCHORED PROTEIN* (n-002), *OsFBX213* (n-006) and *CYCLIN-DEPENDENT KINASE INHIBITOR* (n-006) among others. Thus, expression profiling in the adult tissues of rice plant provided useful insight into the probable functionality of these novel miRNA genes.

In summary, rapid advances in deep sequencing technology has greatly assisted discovery of miRNA genes in rice^14^,^15^,^16^,^58^,^59^,^60^. Identification of 71 novel miRNAs and unreported orthologous miRNAs in rice is significant as it expands the number of biological activities that are under the regulation of miRNAs. Moreover, it also implies that there could be several other miRNAs that remain undiscovered. Thus, further studies should still be carried out on diverse tissue and growth conditions to identify novel miRNA genes in rice. Previous findings have also indicated that there must be a large number of miRNAs that may be unique to the rice genome, and possibly many more miRNAs remain unidentified^60^. Moreover, while further investigations would need to be done, nevertheless, it appears that there could be functional miRNA genes which do not fall within the confines of a classical miRNA gene definition. It would be very interesting to analyze the activity (biogenesis and targeting) of such loci as it may help better understand the diversity of miRNA genes.

## Methods

### Plant growth conditions and stress treatment

Seeds of *Oryza sativa* L. ssp. *aus* cultivar N22 were surface sterilized with 0.1% HgCl_2_ and Teepol, rinsed and then soaked in R.O. water overnight. Seeds were grown on muslin cloth tied over a tray containing Yoshida rice growth medium {40 mg/L NH_4_NO_3_, 10 mg/L NaH_2_PO_4_.2H_2_O, 40 mg/L K_2_SO_4_, 40 mg/L CaCl_2_, 40 mg/L MgSO_4_.7H_2_O, 0.5 mg/L MnCl_2_.4H_2_O, 0.05 mg/L 0.2 mg/L H_3_BO_3_, 0.01 mg/L ZnSO_4_.7H_2_O, 0.01 mg/L CuSO_4_.5H_2_O and 2 mg/L FeCl_3_.6H_2_O (in monohydrate citric acid) with pH adjusted to 5} for two weeks in culture room with 28 ± 2 °C for 16/8 h photoperiod and then transplanted to field. Drought treatment was given to field grown mature plants by withholding water supply 10–15 days before the expected date for ‘heading’. Soil moisture was monitored using Hydra Probe Soil Moisture Sensor. Tissue was collected when soil moisture content reached below 15% and plants showed distinct leaf rolling phenotype. Tissues (flag leaves, spikelets and roots) were collected only from plants that were at an appropriate stage of panicle development (heading or anthesis). Drought induction was checked by estimating the transcript levels of drought stress marker genes *Rubisco small subunit* (*RBCS*)^61^ and *OsbZIP23*^62^ in the collected tissue.

### *In-silico* analysis to identify novel miRNAs

The sRNA tags (19,555,985) from 5 sRNA libraries were mapped to N22 genome sequence (Indica Rice Database; www.genomeindia.org.in/irdb) with the help of ‘maq’ software. Simultaneously, the tags were also mapped to miRBase and Rfam (excluding miRNAs) databases and reads that matched with ≥80% similarity with miRBase (http://www.mirbase.org/) and ≥90% with Rfam database (http://rfam.sanger.ac.uk/) were excluded from downstream analysis. The mapping of 15,933,373 remaining tags was further analyzed to identify loci where significant numbers of tags were mapped within a window size of 5 bases with respect to the 5′ end-mapping coordinate. 2,610,147 such clusters were identified, out of which 145,028 clusters had ≥10 reads per cluster. Further analysis was done on these clusters to identify both intergenic and intronic putative novel miRNA genes. For intergenic novel miRNAs, clusters falling within the CDS, intron and UTR regions of rice genes (RGAP, http://rice.plantbiology.msu.edu/) were removed to represent only the intergenic regions of genome. Flanking 100 bp genomic sequence on either side of such clusters (120,456) was extracted from the N22 genome (Indica Rice Database or IRDB; www.genomeindia.org/irdb). These sequences (~200 bp) were later trimmed down to adequate length capable of forming the hairpin. The secondary structures were generated using UNAFold and mfold softwares^63^. The resultant structure files (.ct) were initially screened with the help of in-house developed perl scripts for a suitable hairpin structure. For finding intronic miRNAs, clusters falling within the introns were retained (57,688) and subjected to similar analysis as described above. It was followed by checking the structures and mapping pattern of the sRNA reads on the precursor sequences to identify the region of high reads accumulation in a non-phasic manner so as to represent the mature sequence. Screening criteria^64^ was followed as (i) read abundance of ≥10 from multiple independent experiments (ii) ability of flanking sequences to form a miRNA precursor-like hairpin with miRNA:miRNA* duplex (iii) mapped reads should originate from non-coding regions (iv) reads annotated as mature miRNA should have a consistent 5′ end while the 3′-end may be more variable (v) preferably presence of tags corresponding to both miRNA and miRNA*. The sample details of all the libraries used in this study are given in Supplementary Table S14.

### Identification of putative orthologous miRNA genes

In order to identify orthologous miRNAs in N22, ‘miFam.dat’ file was downloaded from miRBase 21. Out of 7,057 plant specific precursors, those specific to rice are 592 while the rest 6,465 belong to plants other than rice. 5,099 precursors are distributed among 525 different families while the rest 1,958 are not assigned to any family. The distribution of 525 families is such that 35 are rice specific, 38 are common among rice and other plants while 452 families are not found in rice. With these remaining 452 families (comprising of 3,204 precursors), downstream analysis for ortholog identification was carried out. 3,204 precursor sequences of miRBase were mapped on N22 genome by performing blastn. Twenty-two had ≥50% precursor sequence similarity, which were checked for the conservation of mature sequence region. 16 out of 22 had ≤3 mismatches in the mature region. Secondary structures were generated using RNAfold^65^ (http://rna.tbi.univie.ac.at/cgi-bin/RNAfold.cgi).

### Phylogenetic conservation and divergence of novel miRNAs

Small RNA libraries were downloaded from NCBI SRA database (http://www.ncbi.nlm.nih.gov/sra/) for two dicots, *viz. Arabidopsis thaliana* (SRX058635, SRX058636 and SRX058638) and *Glycine max* (SRX031157 and SRX131086); and four monocots: *Oryza sativa* (ERX014997, SRX017641 and SRX024857), *Triticum aestivum* (SRX131958 and SRX131964), *Zea mays* (SRX015774) and *Hordeum vulgare* (SRR513546, SRR513547 and SRR513548). Trimmed sRNA reads were aligned to the mature novel miRNA sequences.

### Expression profiling on tag density

The expression profile of the novel miRNAs was determined by mapping the sRNA libraries from flag leaf, spikelets (heading and anthesis) and mature roots from control and drought treated N22 plants (Supplementary Tables S11 and S12). The number of tags mapping to each novel miRNA was normalized and the expression values were represented as reads per million (RPM) of the total genome-mapped sRNA tags in the library. The statistical significance of the fold change was calculated as reported earlier^66^,^67^.

### Quantitative RT-PCR

Total RNA was extracted from harvested tissues using TRI Reagent (Sigma) followed by DNaseI treatment (Fermentas). Small RNA samples enriched using 4M LiCl were polyadenylated using Poly(A) Tailing kit (Ambion) and 2 μg of each sample was reversed transcribed with miR_oligodT_RTQ primer for first strand cDNA synthesis using SuperScript II Reverse Transcriptase (Invitrogen). To analyze the expression of the mature miRNAs, qRT-PCR was done using TaqMan Fast Universal PCR Master Mix (ABI) with RTQ universal reverse primer, miRNA specific forward primer and fluorogenic probe^68^ in StepOnePlus Real-Time PCR System (ABI) according to the manufacturer’s protocol. The expression level of miRNA was normalized using 5S rRNA’s expression as an endogenous control. ∆∆Ct method was employed to calculate relative fold change (2^−∆∆Ct^) in expression and standard error was calculated. The detail of primers used for qRT-PCR expression analysis is given in Supplementary Table S15.

### Degradome library preparation

Degradome libraries were prepared^38^ from three different tissues of N22, *viz*. heading spikelet, anthesis spikelet and flag leaf at heading stage. Briefly, polyA enriched mRNAs obtained by using Oligotex kit, (QIAGEN) were ligated with 5′-RNA adapter having an *Mme*I site using T4 RNA Ligase (Ambion) and reversed transcribed with an extended oligo(dT) having a 3′ adapter sequence using Superscript II RT (Invitrogen). A short PCR of 15 cycles was performed to amplify the cDNA and the PCR product was digested with *Mme*I (NEB) to generate equal-sized fragments that was recovered by 12% PAGE. Then a double-stranded 3′-DNA adapter was ligated to the *Mme*I digested products. The resulting product was PCR-amplified (21 cycles), gel-purified, cloned in PUC19 to check the quality of library prepared and then given for high-throughput sequencing by Illumina HiSeq 2000 platform. Details of all the primer and adapters used in degradome library preparation are given in Supplementary Table S15. The degradome data was then analyzed with the help of CleaveLand (ver. 3.1.1) pipeline to identify target genes and generate t-plots, utilizing the mature sequences of sRNAs and cDNA transcripts of RGAP (TIGR 7). As per the analysis, the targets are identified in 5 categories (category ‘0’ to category ‘4’)^39^. Category ‘0’ is the best while category ‘4’ is the least significant. Category ‘0’ indicates the best possible condition in which the cleaved site has the maximum number of degradome reads (>1) and there is only one position in the transcript for this maximum value. Category ‘1’ means >1 read which is equal to the maximum number of reads on the transcript when there is >1 position at maximum value. Category ‘2’ means >1 read which is >average depth but not the maximum on the transcript. Category ‘3’ means >1 read but ≤average depth on transcript while category ‘4’ means a single read at that position. P-value is the indication of statistical significance and is based on the number and distribution of the degradome reads on the target transcript with reference to the miRNA-binding site. Alignment score is based on miRNA-mRNA alignments and is calculated on position-weighted scoring matrix^39^.

## Additional Information

**How to cite this article**: Mutum, R. D. *et al.* Identification of novel miRNAs from drought tolerant rice variety Nagina 22. *Sci. Rep.* **6**, 30786; doi: 10.1038/srep30786 (2016).

## Supplementary Material

Supplementary Information

Supplementary Table S1

Supplementary Table S2

Supplementary Table S3

Supplementary Table S4

Supplementary Table S5

Supplementary Table S6

Supplementary Table S7

Supplementary Table S8

Supplementary Table S9

Supplementary Table S10

Supplementary Table S11

Supplementary Table S12

Supplementary Table S13

Supplementary Table S14

Supplementary Table S15

## Figures and Tables

**Table 1:** List of putative orthologous miRNAs identified in N22.

Parent miRBase	Rice orthologue	Chromosome	No. of mismatches	Mature sequence in N22	RNAfold structural feature
bdi-MIR5056	miR5056	10	0	AGGAAGAACUGGUAAUAAGCA	consistent
bdi-MIR7767	miR7767	11	1	CCCCAAGCUGAGGGCUCUCCC	inconsistent
gma-MIR5368	miR5368	4	0	GGACAGUCUCAGGUAGACA	consistent
hbr-MIR6485	miR6485	10	1	UAGGAUGUAGAAGAUCAUAA	inconsistent
hvu-MIR6183	miR6183	4	1	UGAACGAGUUGGCUGCAAGUUC	inconsistent
hvu-MIR6187	miR6187	1	0	UGAACAGGUUCGGCGACCUCA	consistent
hvu-MIR6194	miR6194	5	3	UAUGGAGACCUGACAGAUGAG	inconsistent
hvu-MIR6196	miR6196	6	1	AGGACGAGGAGAUGGAGAAGA	consistent
hvu-MIR6199	miR6199	5	0	CCACAGAAUUCUCACAGUGAUGG	inconsistent
hvu-MIR6206	miR6206	5	3	GGUACACGUGCUGCAAGCAUAG	inconsistent
hvu-MIR6207	miR6207	2	0	UGGACGACCUGGGCGCCGACG	inconsistent
peu-MIR2916	miR2916	9	2	UGGGGGCUCGAAGACGAUCAGAU	inconsistent
rgl-MIR5141	miR5141	9	1	AGACCCGACGCGACUGACGGAUAA	inconsistent
sbi-MIR6221	miR6221-5p	10	2	UUCUGACCUGUGGCCCCUGCU	consistent
	miR6221-3p		2	CUGGGGCCACAUCUCAGAAGC	
tae-MIR9774	miR9774	10	2	CAAGAUAUUGGGUAUUUCUAGC	consistent
tae-MIR9780	miR9780	3	2	CGGGUCCGCGCUGCACGCCGC	inconsistent

The parent miRBase species, chromosomal location, mature sequence and RNAfold feature are given for each of the 16 MIR genes (17 mature sequences). The parent species are bdi (*Brachypodium distachyon*), gma (*Glycine max*), hbr (*Hevea brasiliensis*), hvu (*Hordeum vulgare*), peu (*Populus euphratica*), rgl (*Rehmannia glutinosa*), sbi (*Sorghum bicolor*), tae (*Triticum aestivum*).

**Table 2:** List of high confidence miRNA:target pairs.

Novel miRNAs	Class	Target LOCs	Reads	Category	P-value	Target gene
n-001	class-I	03g57940.4	11	1	0.01	CK1_CaseinKinase_1a 4 (Hd16)
n-028	class-I	05g02120.1	34	0	0.04	Pectinacetylesterase domain containing protein
n-042	class-II	05g07030.2	38	0	0.02	arginyl-tRNAsynthetase
n-046	class-I	05g41480.1	13	0	0.03	DUF250 domain containing protein
n-098	class-II	08g38620.3	15	0	0.03	expressed protein
		08g38620.3	24	0	0.02	expressed protein
		08g38620.3	45	0	0.03	expressed protein
		09g36320.1	85	0	0.03	tyrosine protein kinase domain containing protein
n-102	class-I	08g38620.3	15	0	0.03	expressed protein
		08g38620.3	24	0	0.02	expressed protein
		08g38620.3	45	0	0.02	expressed protein
n-137	class-II	07g09150.1	14	1	0.04	expressed protein
n-156	class-II	02g52250.1	10	1	0.01	SKIP/SNW domain containing protein
		06g48500.2	21	1	0.02	expressed protein
n-173	class-I	04g19480.1	21	0	0.03	retrotransposon protein
		05g43650.1	22	0	0.03	expressed protein
		12g37510.1	23	0	0.04	UDP-glucoronosyl and UDP-glucosyltransferase domain containing protein
		02g03750.1	25	0	0.01	Polygalacturonase
		05g25890.1	27	0	0.03	expressed protein
		09g30140.1	40	0	0.02	expressed protein
n-199	class-I	08g26230.7	10	0	0.01	expressed protein
		01g49065.1	11	0	0.01	expressed protein
n-220	class-I	06g38080.1	12	0	0.00	von Willebrand factor type A domain containing protein
n-047	class-I	01g44140.1	37	0	0.02	expressed protein
n-054	class-II	09g14560.1	16	0	0.04	expressed protein
n-108	class-II	06g37610.1	16	0	0.01	Cyclopropane-fatty acyl-phospholipid synthase
		06g37610.2	17	0	0.01	Cyclopropane-fatty-acyl-phospholipid synthase
n-118	class-II	09g37240.1	101	0	0.04	glutathione S-transferase, C-terminal domain containing protein
		06g38080.1	12	0	0.04	von Willebrand factor type A domain containing protein
n-139	class-I	07g02330.1	15	0	0.02	protein phosphatase 2C
n-140	class-I	09g27250.1	23	0	0.00	plant viral response family protein
n-200	class-II	02g43110.1	11	0	0.03	sodium/calcium exchanger 1 precursor
n-168	class-I	08g38620.3	24	0	0.04	expressed protein

miRNA:target pairs of novel miRNAs that have significant degradome reads (≥10), category (≤2) and P-value (≤0.05).
