# Supplementary material for: Identification of novel miRNAs from drought tolerant rice variety Nagina 22
Source: Sci Rep. 2016 Aug 8;6:30786. doi: 10.1038/srep30786 (PMC4976344; doi:10.1038/srep30786)
Supplement: Supplementary Information [file srep30786-s1.pdf]

Supplementary Information

Identification of novel miRNAs from drought tolerant rice variety Nagina 22

Roseeta Devi Mutum[1], Santosh Kumar[1], Sonia Balyan[1], Shivani Kansal[1], Saloni Mathur[2] & Saurabh Raghuvanshi[1*]

[1]Department of Plant Molecular Biology, University of Delhi South Campus, Benito Juarez Road, New Delhi – 110021, India

[2] National Institute of Plant Genome Research, Aruna Asaf Ali Marg, New Delhi – 110067, India

*Corresponding author

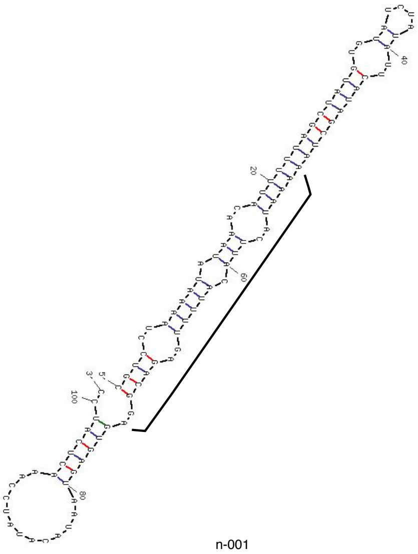

n-001

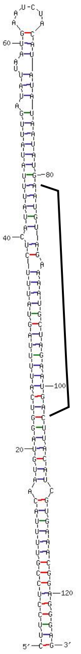

n-002

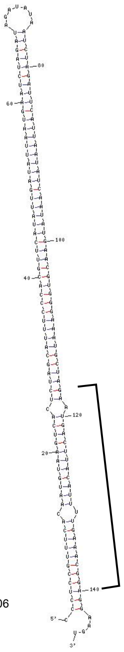

n-006

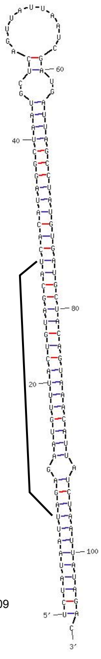

n-009

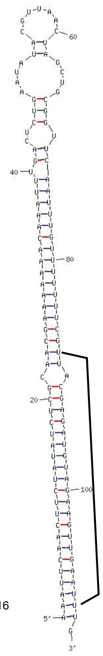

n-016

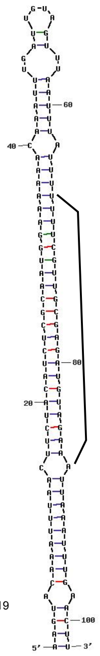

n-019

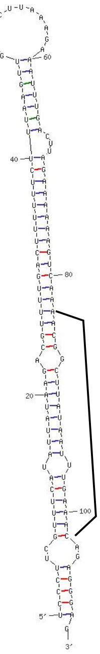

n-020

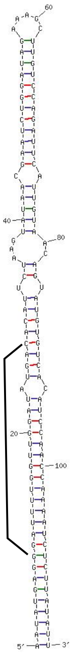

n-024

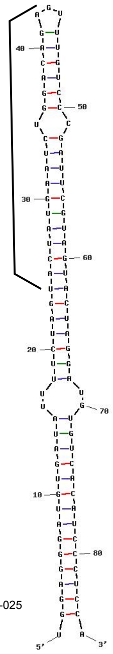

n-025

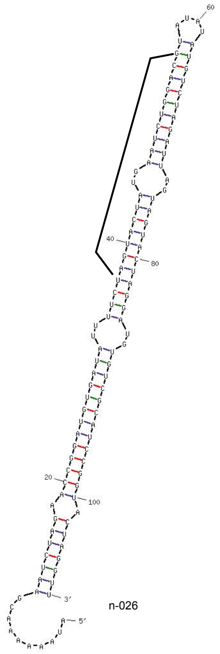

n-026

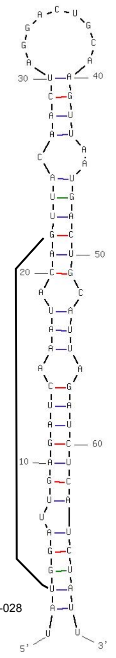

n-028

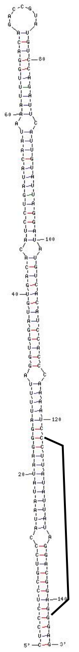

n-032

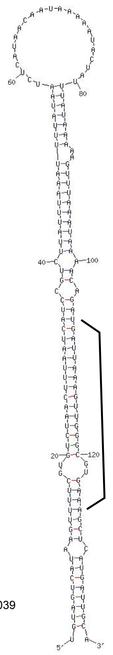

n-039

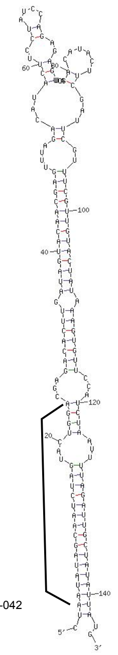

n-042

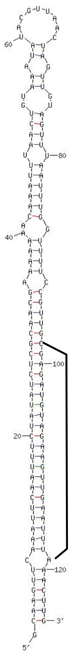

n-046

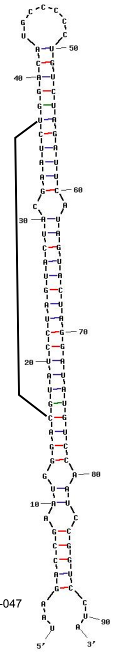

n-047

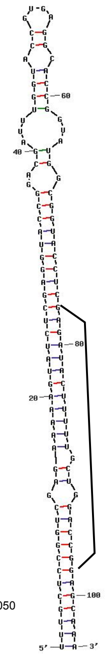

n-050

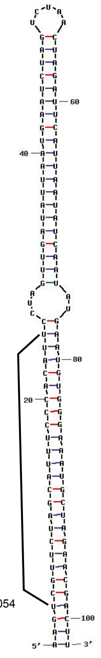

n-054

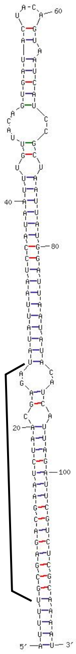
n-055

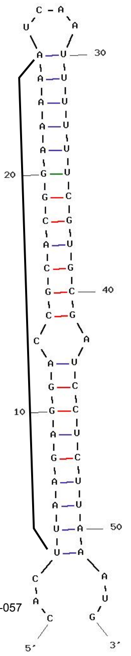
n-057

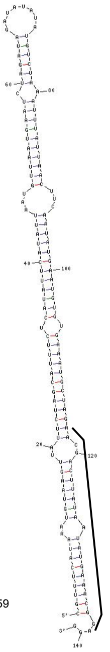
n-059

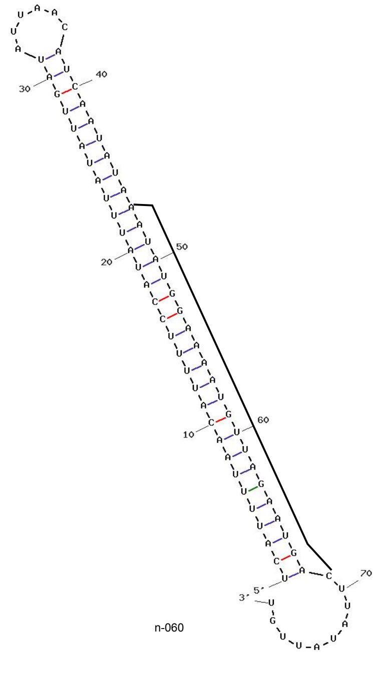
n-060

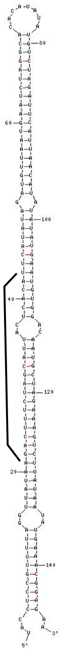
n-063

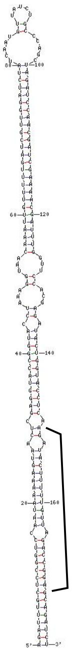
n-064

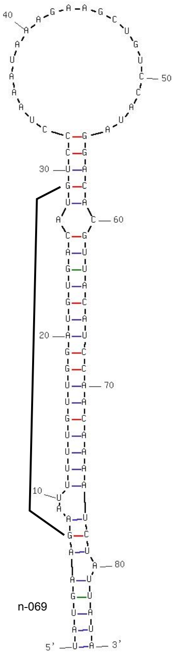
n-069

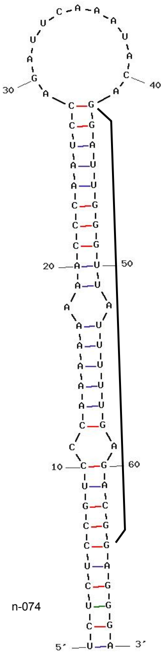
n-074

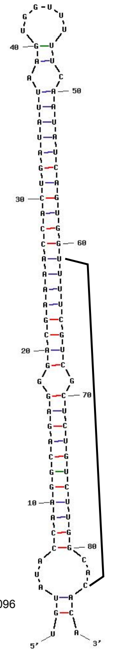
n-096

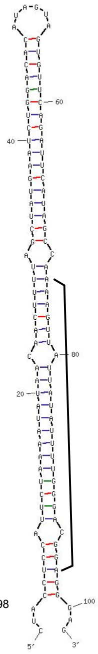
n-098

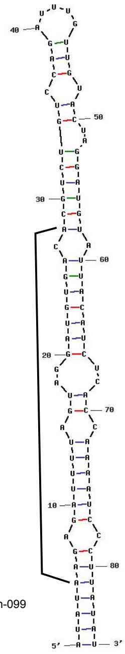
n-099

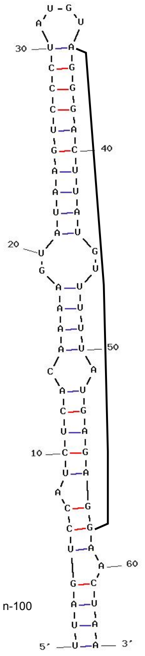
n-100

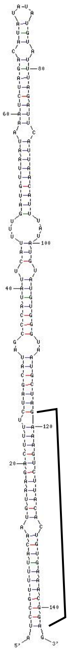
n-102

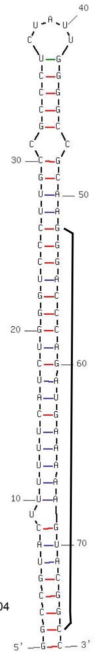
n-104

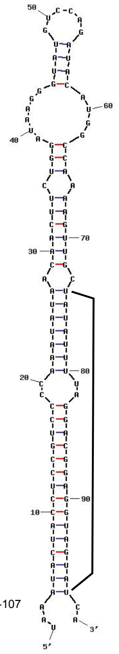
n-107

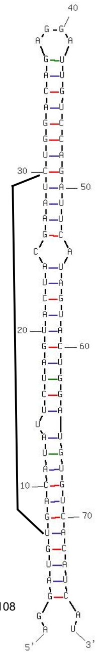
n-108

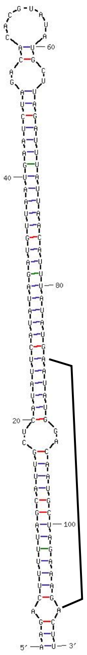
n-113

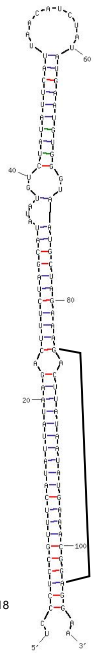
n-118

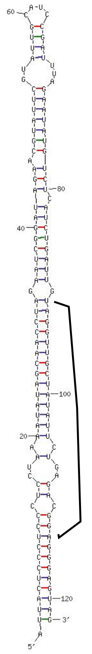

n-120

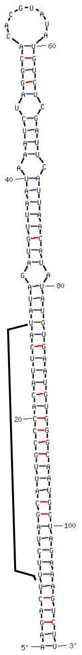

n-121

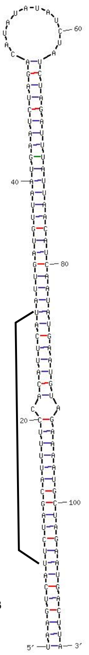

n-123

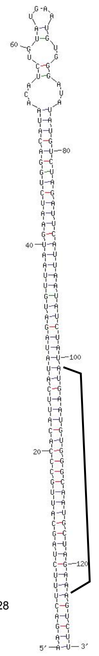

n-128

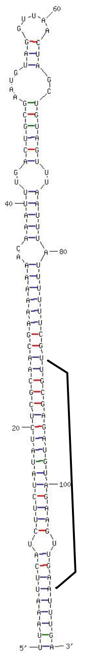

n-129

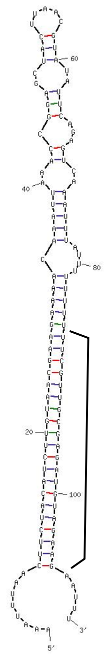

n-130

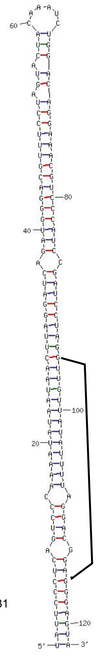

n-131

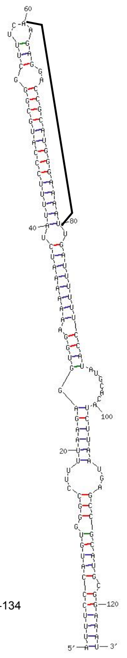

n-134

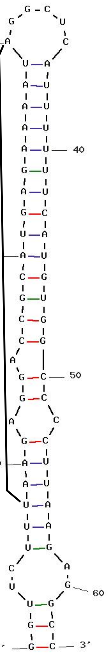

n-135

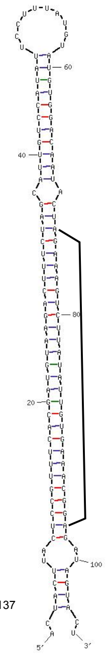
n-137

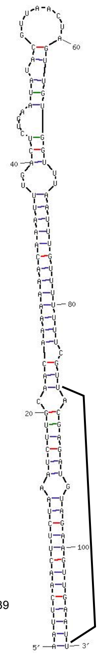
n-139

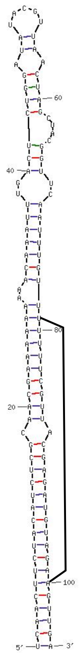
n-140

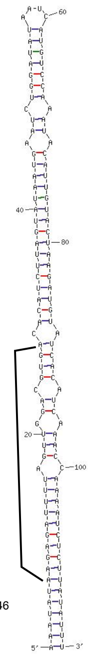
n-146

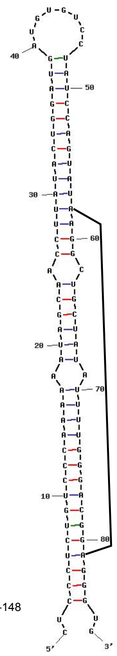
n-148

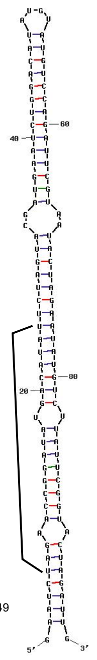
n-149

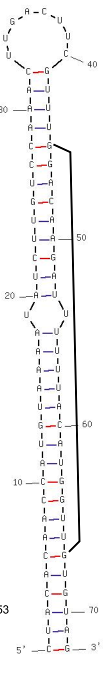
n-153

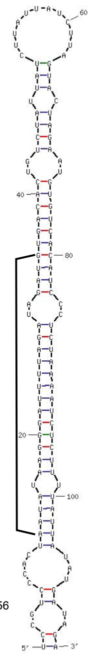
n-156

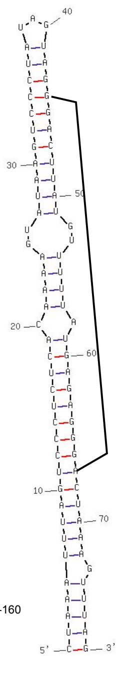
n-160

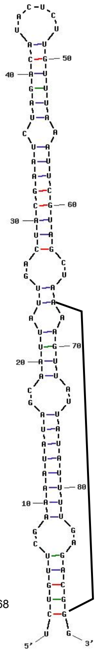

n-168

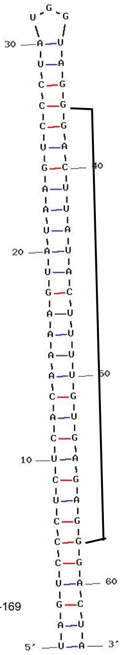

n-169

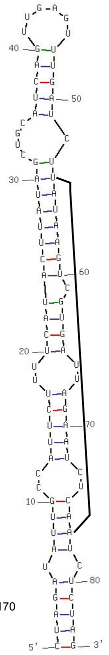

n-170

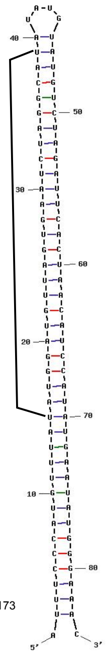

n-173

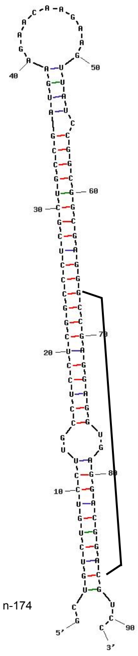

n-174

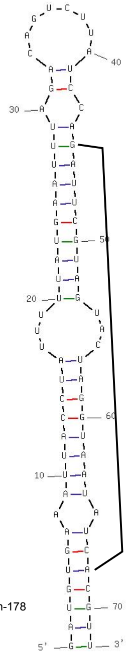

n-178

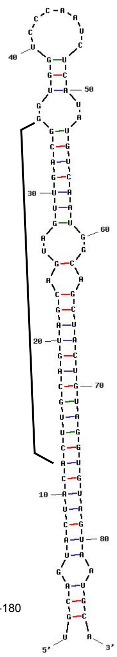

n-180

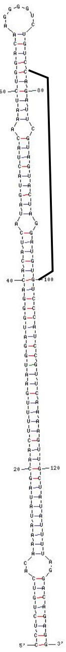

n-184

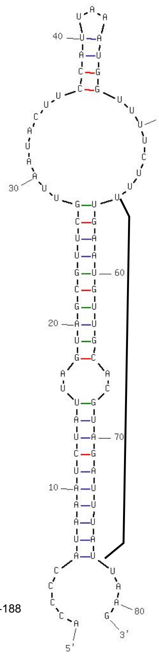

n-188

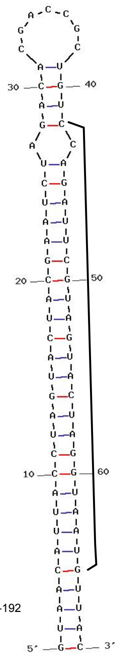

n-192

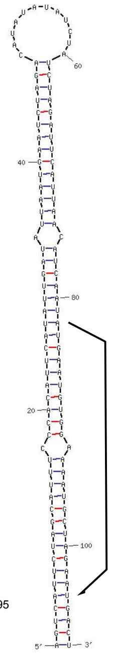

n-195

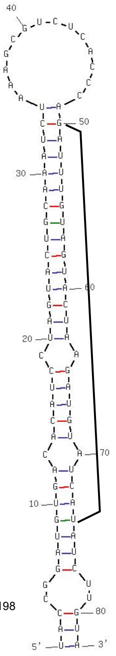

n-198

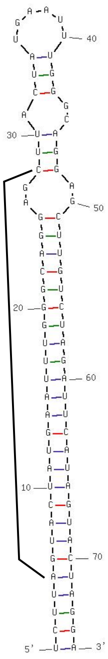

n-199

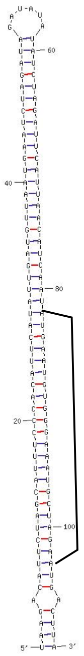

n-200

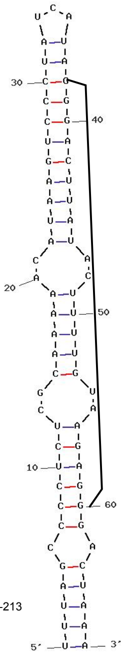

n-213

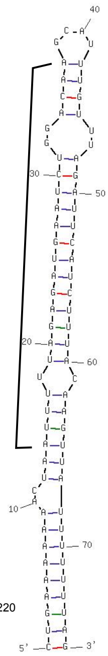

n-220

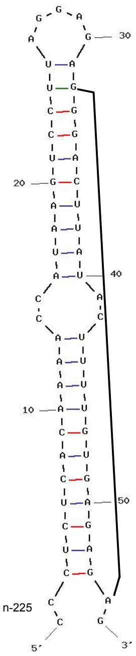

n-225

Fig. S1. Secondary hairpin structures of 71 novel miRNAs.

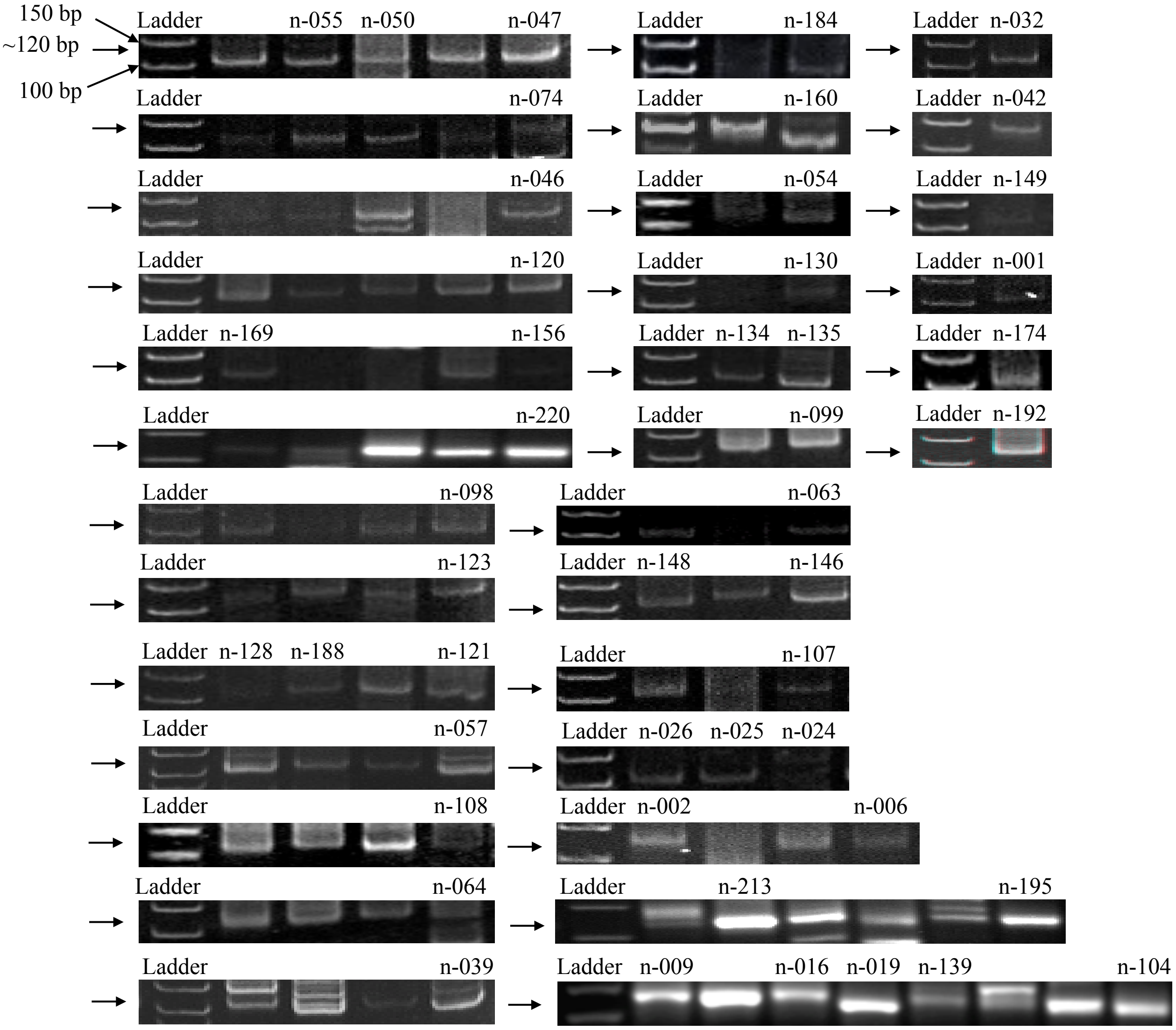

Fig. S2 . Detection of novel miRNA candidates. Gel profile of 47 novel miRNAs (products of qRT-PCR loaded on 6% PAGE gel). Amplified product includes ~20 bp of miRNA + 100 bp of adapter and the arrow indicates the size of ~120 bp.

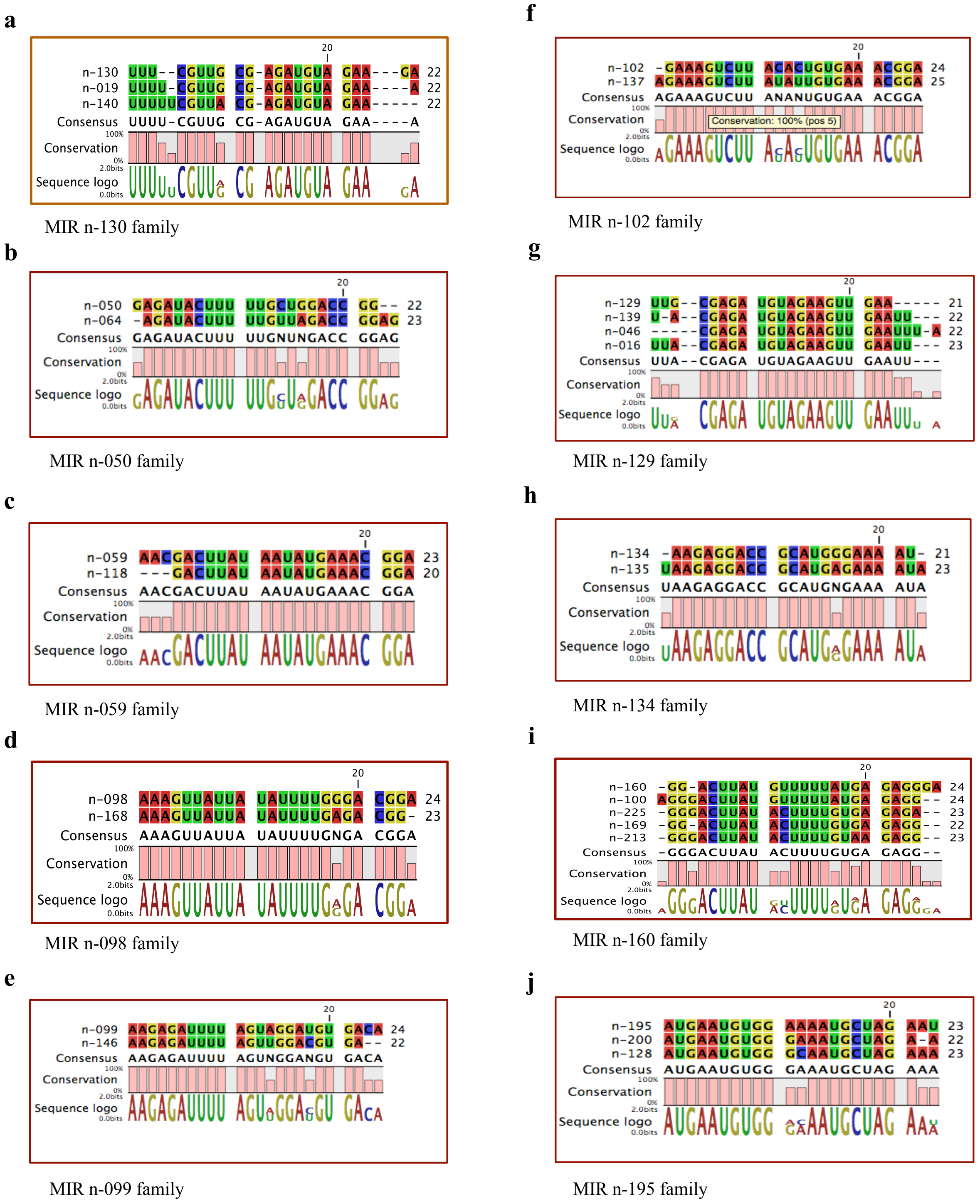

Fig. S3. Multiple sequence alignment among family members of novel miRNAs. 27 miRNAs are distributed among 10 families based on mature sequence conservation. Alignments for MIR families (**a**) n-130 (**b**) n-050 (**c**) n-059 (**d**) n-098 (**e**) n-099 (**f**) n-102 (**g**) n-129 (**h**) n-134 (**i**) n-160 (**j**) n-195 are shown.

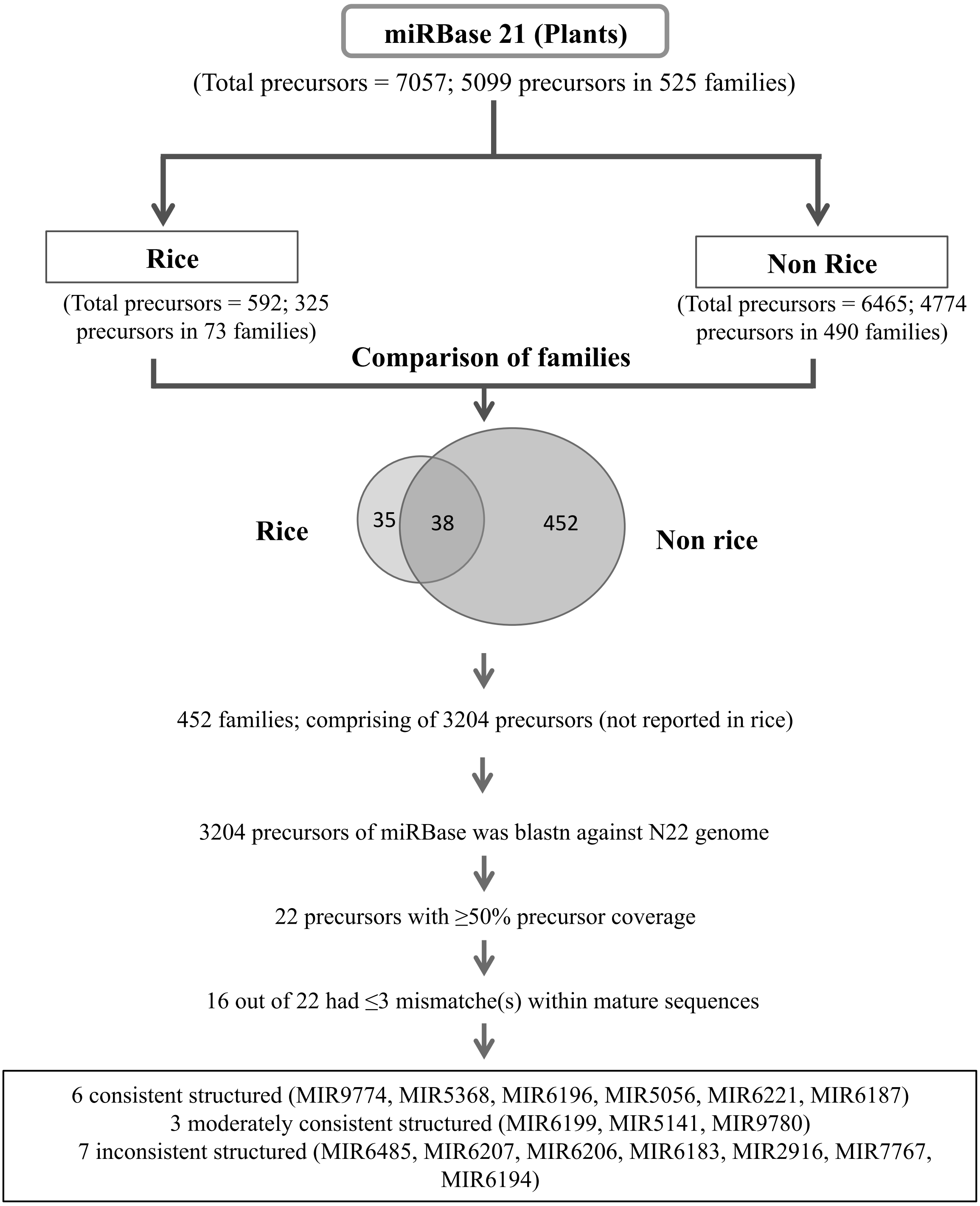

Fig. S4. Flowchart for identification of putative orthologous MIR genes in N22. Sixteen putative orthologous MIR genes are identified in N22 comprising of 6 consistent and 10 inconsistent structures.

```
tae-MIR9774       AAGACAGAAAUACCCAAUAUCUUGCUAGAACAAGAUAUUGGGUAUUUCUGUCUU
osa-MIR9774       AAAAAAGAAAUACCCAAUAUCUUGCUUCAGCAAGAUAUUGGGUAUUUCUAGCUU
                  ** * *********************  * *******************  ***

hvu-MIR6199       AAGCUUGCAGCCACAGAAUUCUCACAGUGAUGGGCCUCCUCUUGCCCCGUGUCAGUCAAG
osa-MIR6199       GAGUCUGCUGCCACAGAAUUCUCACAGUGAUGGGCUACCCCUUGCCCCAUGUCAAUCAAG
                   **  *** **************************  ** ******** ***** *****

                  GCCAUGGUGGUCAAAGCCAUCCUUUCUUAUGGAUCAACAGCUUCAUCCUGUGCCAUCAAC
                  GCCAUGGUGGUCAAAGCCAUCAUUCCUUAUGGAUCAUCAGCUUCAUCCAGCUCCUUCAAC
                  ********************* ** *********** *********** *  ** *****

                  UGUGACAAAUUCUGUGGUCAGUAGGAUA
                  UGUAACUAAUUCUGUAGUCAGUAGGAUA
                  *** ** ******** ************

hbr-MIR6485       ACCUAGGAUGUAGAAGAGCAUAACAUGAGUGAUUCAGCAGAUUUGGAUUC-----CUAUA
osa-MIR6485       ACCUAGGAUGUAGAAGAUCAUAACAUGAGCGAUUCGGCAGAUUUGGAUUCCUUUCCUAUA
                  ***************** *********** ***** **************     *****

                  UAUCCACUCAUGUAGUACUUUAUUUCAUUUUACGA
                  UAUCCACUCAUGUGGUACUU-----CAUCAUACGA
                  ************* ******     ***  *****

gma-MIR5368       ACUGUUUCCUGGGAUUGGCUUUGGG-CUUUCCUGCACAGCUUAGGUGGAGGGCAAAGAAG
osa-MIR5368       ACUGUUCCCUGGGAUUGGCUUUGGGCCUUUCCUGCGCAGCUUAGGUGGAAGGCGAAGAAG
                  ****** ****************** ********* ************* *** ******

                  ACUUCCUUCUGAGGGGGCCAGAGCCAUCAGAGAGAUACCACUCUGGACGAGCUAGAAUUC
                  GCCCCCUUCCGGGGGGGCCCGAGCCAUCAGUGAGAUACCACUCUGGAAGAGCUCGGAUUC
                   *  ***** * ******* ********** **************** ***** * ****

                  UAACCUUGUGCCAGGACCUAUGGGCCGAGGGACAGUCUCAGGUAGACAGU
                  UAACCUUGUGUCAG-ACCCGCGGGCCAAGGGACAGUCUCAGGUAGACAGU
                  ********** *** ***   ***** ***********************

hvu-MIR6207       CACGAGGGUGGGCCACAUGGGCGUGAAGGUGGCCAAGUCCAUGGGCCACCACGUGACGGU
osa-MIR6207       CGGCGGCGUGGGGCACAUGGGCGUGAAGGUGGCCAAGUCGAUGGGGCACCACGUGACGGU
                  *    * ***** ************************** ***** **************

                  GAUCAGCUCGUCCAACAAGAAGCGGGCCGAGGCCGUGGACGACCUGGGCGCCGACGAGUA
                  GAUCAGCUCGUCGGCGAGGAAGCGCGGCGAGGCCAUGGACGACCUGGGCGCCGACGCCUA
                  ************    * ****** * ******* *********************  **

                  CCUCGU
                  CCUCGU
                  ******

hvu-MIR6196       CGAGCGAUGGAGGACGAGGAGAUGGAGAGGAAGGUGCAGCAGUACCUGCACCGCAAGGGA
osa-MIR6196       GGCACCAUGGAGGACGAGGAGAUGGAGAAGAAGGUGCAGCAGUACCUGCAGCGGAAGGGG
                   *  * ********************** ********************* ** *****

                  UUCCGCCUCACCGAGCUCGCGCUGCAGGAGGAGCGCAACCGCCUCUCCACCACCUCCCUC
                  UUCCGCCUCACCGAGCUCGCGCUGCAGGAGGAGCGCAACCGCAUCUCCACCUCCUCCGUC
                  ****************************************** ******** ***** **

                  UCCGACGUCUCGCUCU
                  UCCGACGUCGCGCUCG
                  ********* *****

hvu-MIR6206       CGAGGCUGUGGUUCUGCAUCUCCGUGCCACUCACUCUUAUUGGAGGCUUGCUAGGCACAC
osa-MIR6206       UGGCCCUGUGGUUCUGCAUCUCAGUUCCACUCACACUUAUUGGAGGUUUGCUUGGUACAC
                   *   ***************** ** ******** *********** ***** ** ****

                  GGGCUGCAGGCAUAGAAUUCCCUGU
                  GUGCUGCAAGCAUAGAGUACCCUGU
                  * ****** ******* * ******

bdi-MIR5056       CUCAAUUUGGGAGGAAGAACCGGUAAUAAGCAAAAAACCAUCCAUUCAGGUUCUACAUUU
osa-MIR5056       CUUAAUUCGGGAGGAAGAACUGGUAAUAAGCACAAAACCAUCCAUUCUGGUUCUACAUUU
                  ** **** ************ *********** ************** ************

                  GUUUGAAUAAAAUGUUUCGCCAAUUGCAUGCGUCUAAUCAAAAAAACUUUUCUUAUUCAU
                  GUUUGAAUAAAAUGUUUCGCCAAUUGCAUGCGUCUAAUCAAAAAAACUUUUCUUAUUCGU
                  ********************************************************** *

                  CUUUUUCUAUCUUCCCAUUCAUCU
                  CUUUUUCUAUCUUCCCAUUCAUCU
                  ************************
```

```
hvu-MIR6183      GACAAAUUAUUGAGCGAGUUGGCUGCAAGUUCUGAAGUUAAUGAUAAUGGAGUCAGCUCG
osa-MIR6183      GAGAAAUUAUUGAACGAGUUGGCUGCAAGUUCCGAAGUUAAUGACAAUGGCAUCAGCUCC
                 ** ********** ****************** *********** *****  ******* 

                 UUCGUCAUUGCUGUCA
                 UUUGUCAUUGCUGUCA
                 ** *************

peu-MIR2916      UGGGGACUCGAAGACGAUCAUAUACCAUCCUAGUCUCAACCAUAAACGAUGCCGACCAGG
osa-MIR2916      UGGGGGCUCGAAGACGAUCAGAUACCGUCCUAGUCUCAACCAUAAACGAUGCCGACCAGG
                 ***** ************** ***** *********************************

                 GAUUGGCGGAUGUUGCUUCUAGGAGUCCGCCA
                 GAUCGGCGGAUGUUGCUUAUAGGACUCCGCCG
                 *** ************** ***** ****** 

rgl-MIR5141      UUGAGUAAUGCCUGAGUCGCAGCAUCAAGGUCUGAGCCAAAUUGAGCAAAGGCGGCCACU
osa-MIR5141      UUGAGUAAUGCCUGAGUCGCAGCAUCAAGGUCUGACCCAAAUUGAGCGAAGGCGGCCACU
                 *********************************** *********** ************

                 UCGCGAUAUUGUGCCAAUUCCAGUUUUGAACUACCGCAUACUUGUUUCAUAGCUUUCAAC
                 UCGCGAUAUUGUGCCAAUUCCAGUUUUGAACUACCGCAGACUUGUUUCAUAGCUUUCAAC
                 ************************************** *********************

                 UGAGCGGCAGACCCGACGCGACUGACAGAUAAGCCGACGUUA
                 UGAGCGGCAGACCCGACGCGACUGACGGAUAAGCCAACGUUA
                 ************************** ******** ******

bdi-MIR7767      UUGAUGGCUGCCCCAAGCUGAGAGCUCUCCCACGACAGCUUGGACAGGAGGCCACCUGCU
osa-MIR7767      UUGAUAACUGCCCCAAGCUGAGGGCUCUCCCACAACAGCUUGGACACGAGGCCACCAGUU
                 *****  *************** ********** ************ ********* * *

                 UGGAAGAGCUCGGAUUGAGAGGAGCAAGCAGCUUGAAGGUGGUGGAGGACC
                 UGAAAGAGCUCUCCCUUGUAGCAGCAAGCUGCUUGAAGUCGGUGAAGGACC
                 ** ********    *   ** ******* ********  **** ******

hvu-MIR6194      CGCCCACUUUGGGUUUCAGAGAUC------AACG-CCCAUAUAUGCCGCAUGUCAGCCUC
osa-MIR6194      ------AUUGAUGAUUCAU-GAUCUGCUUCAUUGCAGCAUAUAUGCCACACGUCAGCCUC
                        **   * ****  ****      *  *   ********** ** *********

                 CUGUAUGGGGAUCUGACAGACGAGGAGAAGGAAG
                 UUAUAUGGAGACCUGACAGAUGAGGAGAAGGAGG
                  * ***** ** ******** *********** *

sbi-MIR6221      ACAGCUCACCAGCCCAUCCUUAUGCGUGGUGCUUCUGACUUCUGGCCCCUGCUCAGCCCC
osa-MIR6221      ACAGCUCGGCAGCACAGCUUUGUACAUUGUGCUUCUGACCUGUGGCCCCUGCUCAGCCCC
                 *******  **** ** * ** * * * *********** * ******************

                 ACCAACACUAAGCCGGGGCCAGAUCUCAGAAGCAUUGUGCAUAAUCAUGUGCCUCUAGCU
                 ACGGAAAGUAAGCUGGGGCCACAUCUCAGAAGCAUUGUGCAUAAUCCUGUGCUGCUAGUU
                 **  * * ***** ******* ************************ *****  **** *

                 GU
                 GU
                 **

tae-MIR9780      CGCCGUGGUGGCCGAGGAUGCAAGGCGUGGGGGAGCUGGCCAAGGCGUGCACCACCAUCA
osa-MIR9780      CGACGUGGUGGCCGGAGAUGAAGACGGUGGCGGAGCUGGUCAAGGCGUGCGCCACCAUCA
                 ** ***********  **** *    **** ******** ********** *********

                 UCUGGAUCGGGUCGGCGCUGCACGCGGCG
                 UCUGGAUCGGGUCCGCGCUGCACGCCGCC
                 ************* *********** ** 

hvu-MIR6187      ACCGUCGGCAUGAACAGGUUCGGCGACCUCAACCCCAGCGAGGUCGUCGCGCAGUUCACA
osa-MIR6187      UUCGUCGGCAUGAACAGGUUCGGCGACCUCACCCCGGGCGAGUUCGCCGAGCAGUUCACC
                   ***************************** ***  ***** *** ** ********* 

                 GGUUCCAACAACACCGGCUUC-
                 GGGUUCAACGCCGCU-GCUCCA
                 ** * ****  * *  *** * 
```

Fig. S5. Pairwise alignment of precursor sequences of miRBase MIR genes and orthologous MIR genes from N22. Mature sequences are highlighted in yellow and green color in the precursor sequences of parent and N22, respectively. ‘*’ denotes the conserved nucleotides while mismatched nucleotides are indicated by space, ‘-’ denotes gaps.

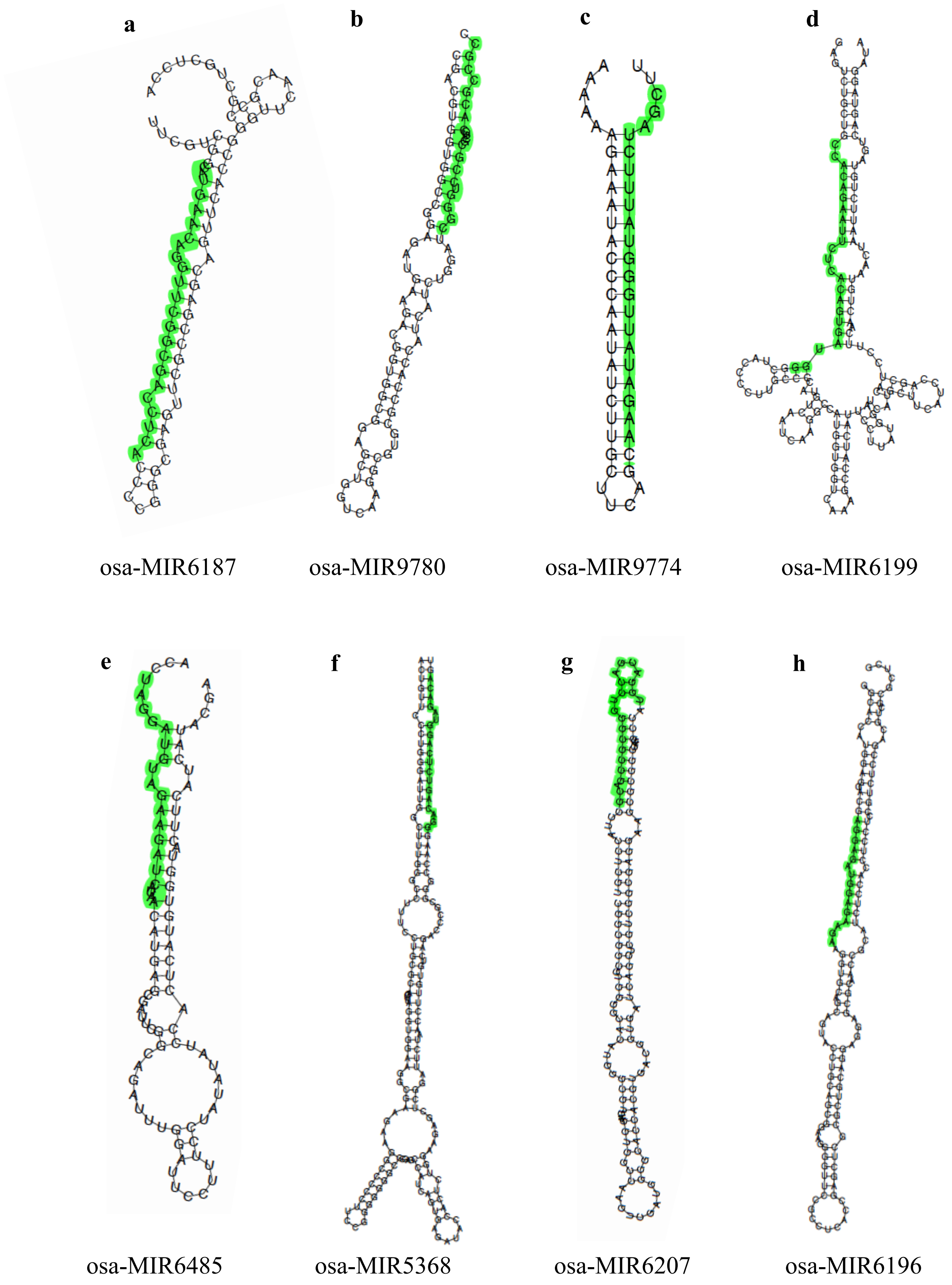
a
b
c
d
osa-MIR6187
osa-MIR9780
osa-MIR9774
osa-MIR6199
e
f
g
h
osa-MIR6485
osa-MIR5368
osa-MIR6207
osa-MIR6196

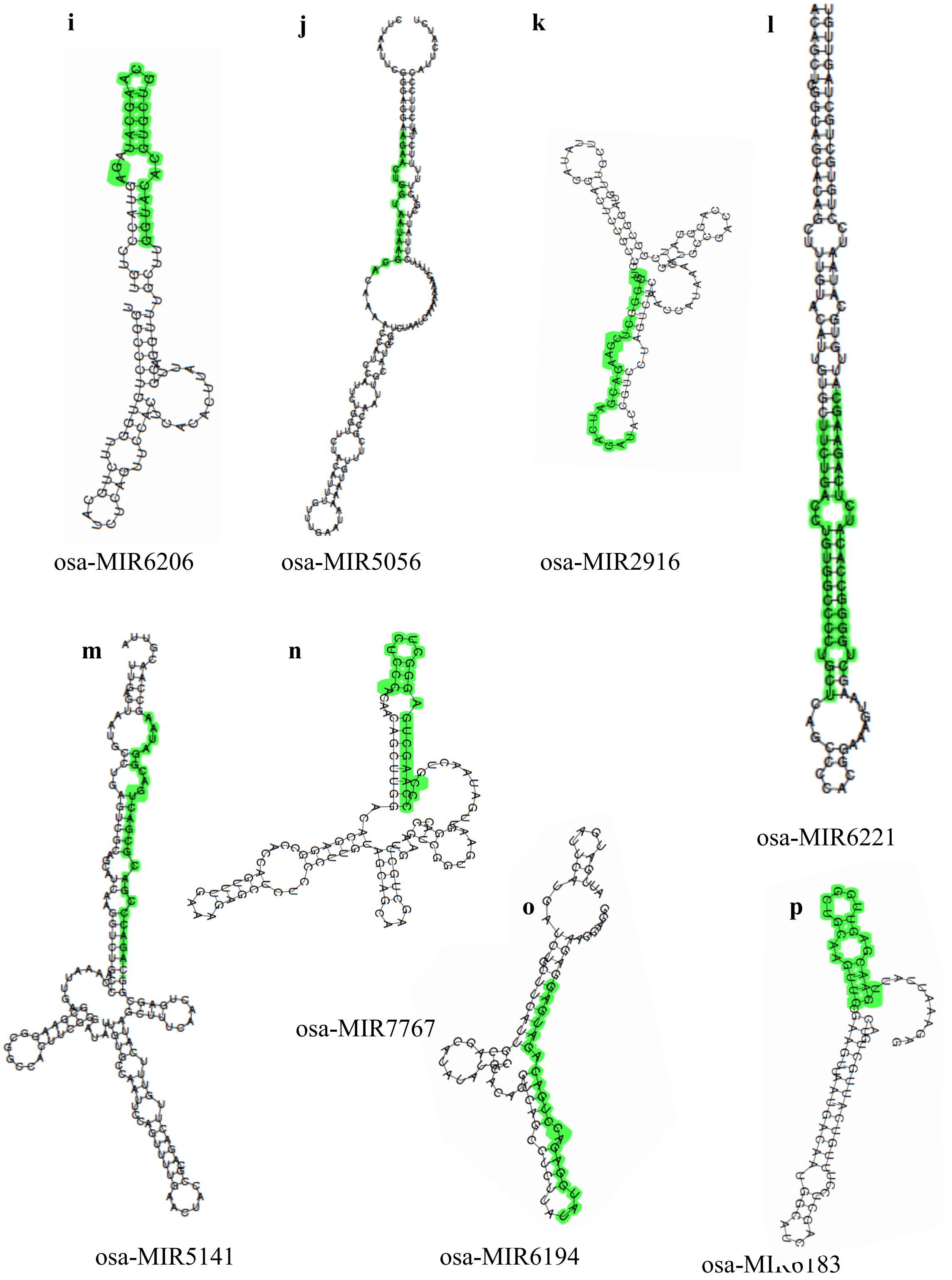

Fig. S6. Secondary hairpin structures of 16 putative orthologous MIR genes in N22. Secondary hairpin foldbacks of (**a**) MIR6187 (**b**) MIR9780 (**c**) MIR9774 (**d**) MIR6199 (**e**) MIR6485 (**f**) MIR5368 (**g**) MIR6207 (**h**) MIR6196 (**i**) MIR6206 (**j**) MIR5056 (**k**) MIR2916 (**l**) MIR6221 (gives rise to both -5p and -3p) (**m**) MIR5141 (**n**) MIR7767 (**o**) MIR6194 and (**p**) MIR6183.

a

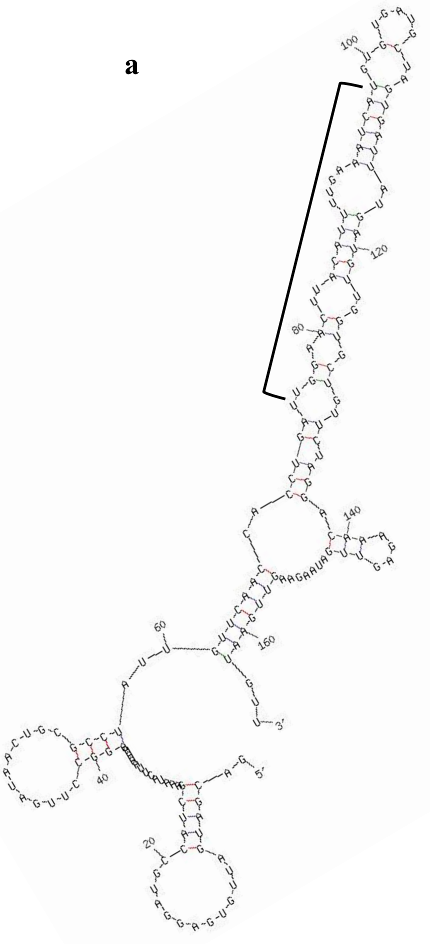

n-006-barley

c

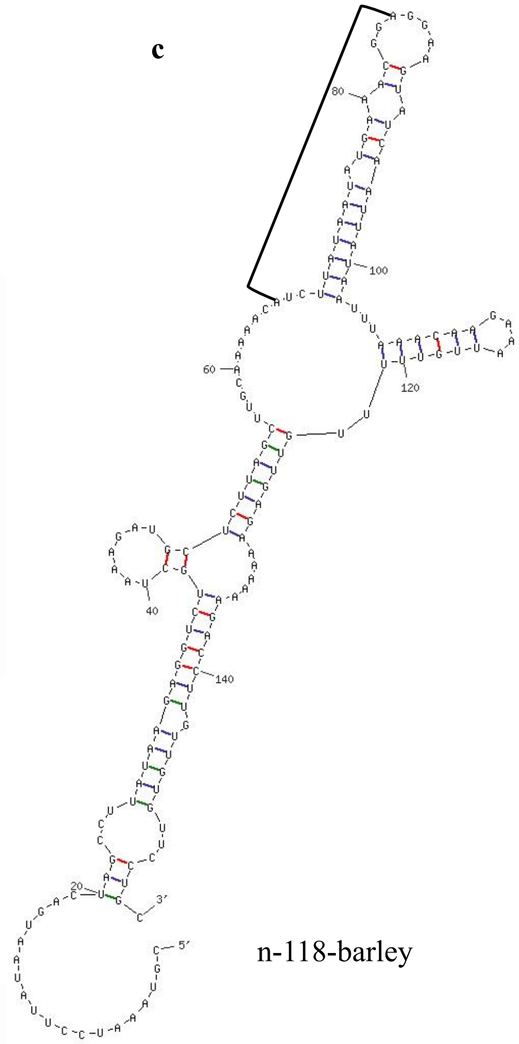

n-118-barley

b

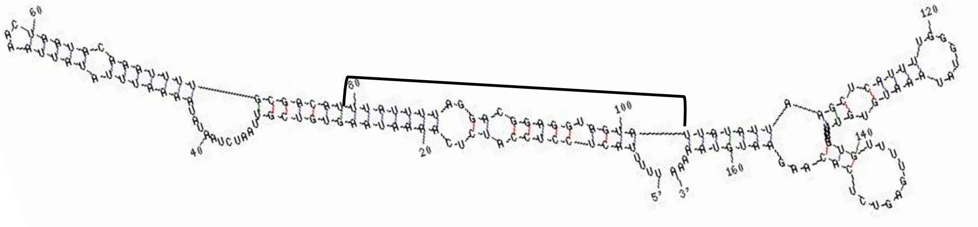

n-107-barley

d

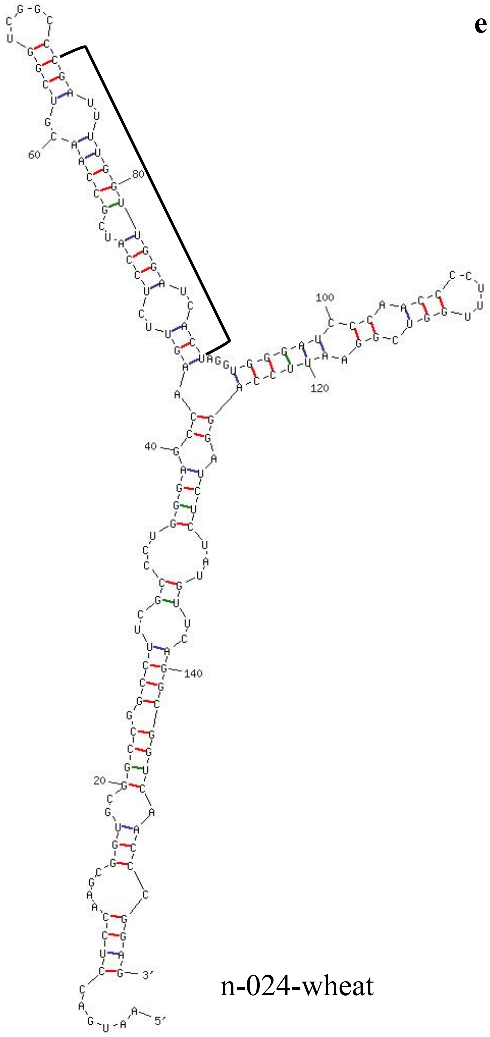

n-024-wheat

e

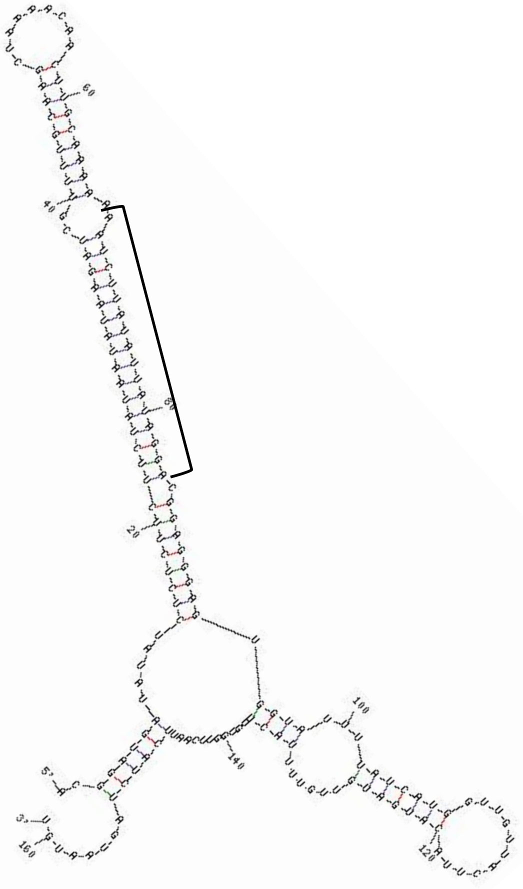

n-032-wheat

f

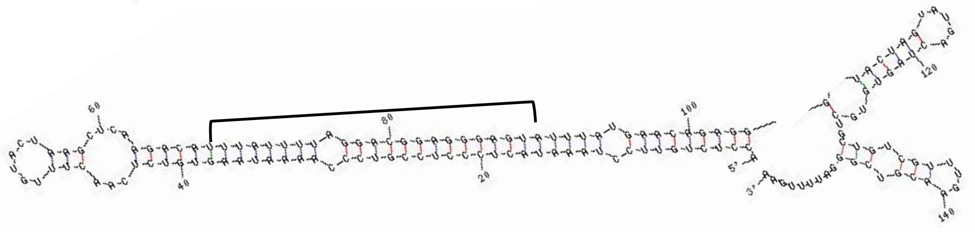

n-107-wheat

Fig. S7. Secondary hairpin structures of the novel orthologous miRNA loci in other plants. Secondary hairpin structures of (**a**) n-006, **(b)** n-107, (**c**) n-118 in barley (**d**) n-024, (**e**) n-032, (**f**) n-107 in wheat.

a

	Degradome reads ≥ 10	Category ≤ 2	p-value ≤ 0.05	Degradome reads ≥ 10, category ≤ 2 and p-value ≤ 0.05
No. of orthologous miRNA:target pair	10:38	15:450	7:10	1:1

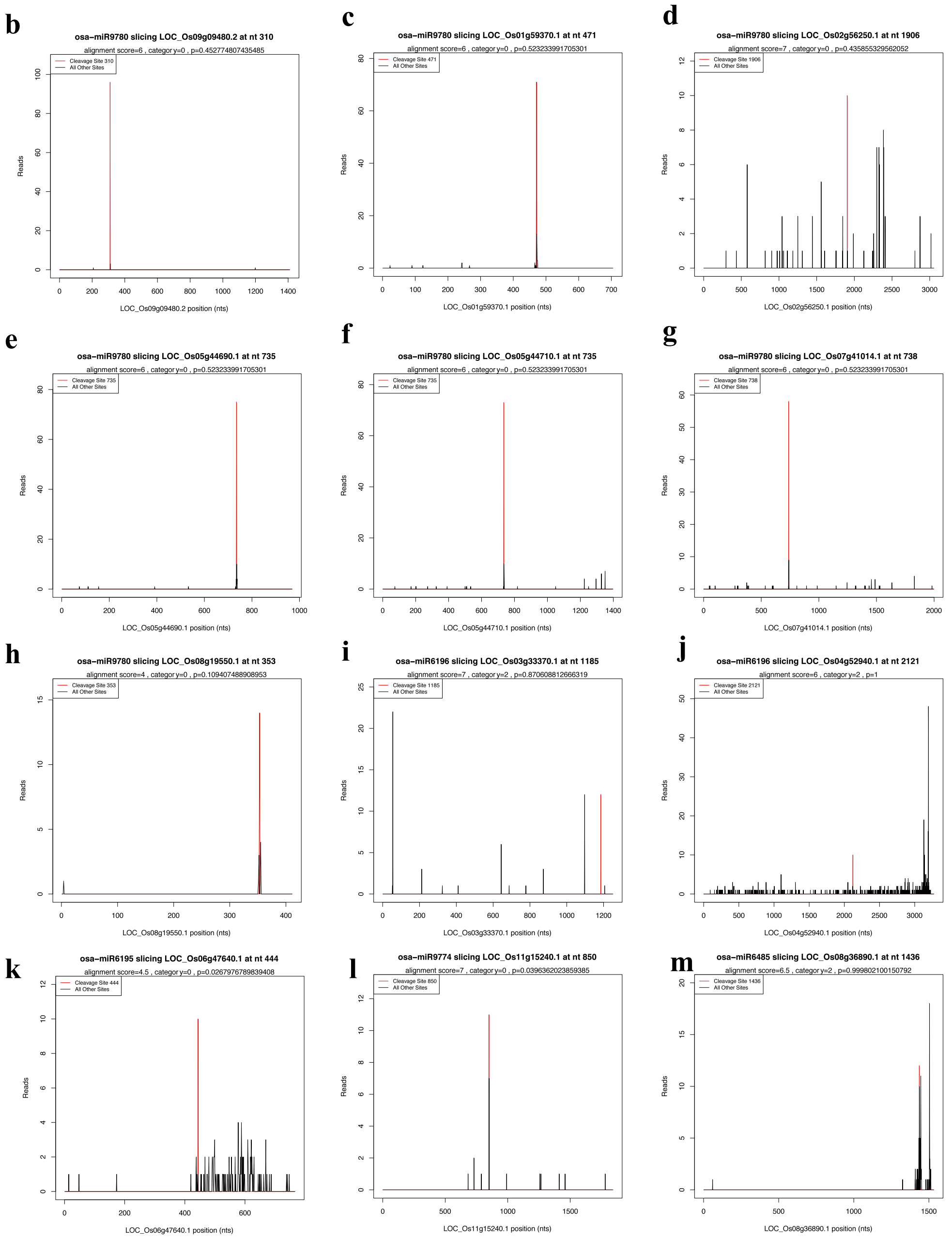

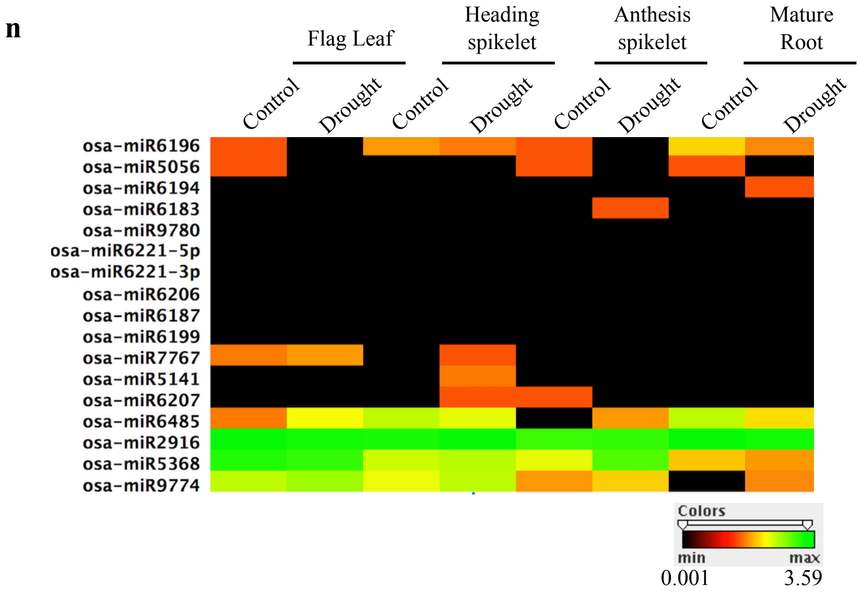

Fig. S8. Characterization of orthologous miRNAs. (**a**) Table showing number of miRNA:target pairs for orthologs based on different PARE criteria. T-plots of osa-miR9774 cleaving (**b**) LOC_Os11g15240, (**c**) LOC_Os01g59370 (**d**) LOC_Os02g56250 (**e**) LOC_Os05g44690 (**f**) LOC_Os05g44710 (**g**) LOC_Os07g41014 (**h**) LOC_Os08g19550 (**i**) osa-miR6196 cleaving LOC_Os03g33370 and (**j**) LOC_Os04g52940 (**k**) osa-miR6195 cleaving LOC_Os06g47640 (**l**) osa-miR9774 cleaving LOC_Os11g15240 (**m**) osa-miR6485 cleaving LOC_Os08g36890. The vertical red line indicates the site of cleavage. (**n**) Heat map of orthologous miRNAs in 8 N22 sRNA libraries.
